# Forecasting regional carbon prices in china with a hybrid model based on quadratic decomposition and comprehensive feature screening

**DOI:** 10.1371/journal.pone.0326926

**Published:** 2025-06-30

**Authors:** Yaoyang Yi

**Affiliations:** School of Finance, Anhui University of Finance and Economics, Bengbu, China; Newcastle University, UNITED KINGDOM OF GREAT BRITAIN AND NORTHERN IRELAND

## Abstract

In light of global climate change and the objective of carbon neutrality, the carbon market has become an important tool for the international community to combat climate change. Nonetheless, due to the complexity and non-linear nature of the carbon price, its accurate prediction has always been a research difficulty. This work presents a hybrid model incorporating comprehensive feature screening, optimized quadratic decomposition, and the Optuna-Attention-LSTM prediction method, aiming to improve the accuracy and stability of carbon price prediction. First, the improved complete ensemble empirical mode decomposition with adaptive noise (ICEEMDAN) is used to decompose the carbon price time series once, extract high-frequency and low-frequency components, and denoise the high-frequency components using stacked denoising autoencoder (SDAE). Then, the variational mode decomposition (VMD) method is subsequently employed to execute a secondary decomposition on the reconstructed signal, with the decomposition hyperparameters optimized via crested porcupine optimization (CPO). Subsequently, Boruta and least absolute shrinkage and selection operator (Lasso) regression are employed to identify significant external features; finally, a long short-term memory (LSTM) model integrated with an attention mechanism is utilized for prediction, and optuna is introduced to optimize the hyperparameters. This paper evaluates the performance of the proposed model using the carbon markets of Guangdong, Hubei, and Shanghai in China as examples. The experimental results indicate that compared with the traditional model, the proposed model achieves average reductions of 67.30%, 47.68%, 48.42%, and 48.79% in the mean squared error (MSE), root mean squared error (RMSE), mean absolute error (MAE), and mean absolute percentage error (MAPE), respectively, demonstrating higher prediction accuracy and robustness. Shapley additive explanations (SHAP) analysis results indicate that carbon prices in the Guangdong carbon market are dominated by macroeconomic and regional environmental factors, while those in the Hubei carbon market are mainly driven by changes in the energy market. The Shanghai carbon market, on the other hand, is more significantly influenced by global carbon market dynamics and international trade activities. The research not only verifies the efficacy of the decomposition ensemble prediction framework, but also provides a scientific basis for decision-making for carbon market participants and policymakers.

## 1. Introduction

The swift progression of industrialization and the extensive use of traditional fossil fuels have further exacerbated the greenhouse effect, with far-reaching impacts on the energy mix and human society [1]. In response to global warming and environmental degradation, countries have put forward targets and commitments to achieve carbon neutrality through policy announcements and executive orders. 1997 saw the adoption of the Kyoto Protocol, which for the first time brought greenhouse gas emissions under legal constraints, marking the birth of the global carbon market [2]. The Paris Agreement adopted in 2015 not only continues the legal framework of the Kyoto Protocol, but also draws on the successful experience of the European Union’s carbon market, marking the beginning of a new phase in global climate governance [3].

To this end, China has introduced a dual carbon objective to attain peak carbon emissions by 2030 and realize carbon neutrality by 2060 [4]. China’s carbon market is currently in a transition stage from early to mature development. Market mechanisms are still imperfect, and relevant laws, regulations, and policy systems need to be strengthened. Since 2011, nine pilot carbon emission trading programmes have been launched in Beijing, Tianjin, Shanghai, Chongqing, Guangdong, Hubei, Shenzhen, Sichuan, and Fujian. As a developing carbon trading market, it is vulnerable to influences such as weather, energy prices, and international carbon prices [5]. These factors interact with each other, increasing the complexity of traditional modeling and forecasting. Therefore, an appropriate feature selection method is crucial in carbon price forecasting. It can not only enhance the precision of predictions, but also improve the model’s interpretability [6]. In addition, the highly accurate decomposition and integration prediction framework can effectively track evolving patterns and operational dynamics of carbon price, enhancing the ability of feature extraction, representation and learning, which enhances predictive performance [7].

### 1.1. Literature review

#### 1.1.1. Forecasting methods in the carbon market.

As carbon trading systems are complex mechanisms, a common theme of discussion in the wider literature is how to enhance the precision of carbon market forecasts and the interpretability of the results. In recent years, carbon market forecasting methods have been classified into three main categories: econometric models, artificial intelligence models, and hybrid models. Conventional statistical and econometric methods are extensively utilized for time series forecasting. For example, Liu et al. [8] found that the GARCH-MIDAS model combining economic policy uncertainty (EPU) and realized volatility (RV) is more effective in predicting China’s carbon volatility than the model based on RV only. Niu and Liu [9] studied the volatility of EU quota futures using the mixed data sampling method based on the GJR-GARCH model. They found that accounting for the asymmetric effect of volatility and the influence of macroeconomic variables can substantially enhance the model’s predictive capability. In addition, Zhang et al. [10] extended the classical autoregressive model and found that the EPU index possesses substantial long-term predictive power for the volatility of EU carbon allowance (EUA) futures.

Although traditional econometric models can intuitively measure the importance of characteristics, they falter in capturing the non-linear aspects of carbon prices. Artificial intelligence models not only have the arithmetic calculation capabilities of traditional econometric models, but also can capture historical information, volatility and nonlinear characteristics. Therefore, they significantly improve the accuracy and performance of predictive analysis. Their advantages in predicting financial time series have now been widely proven [11]. Xu et al. [12] used two traditional econometric models alongside three machine learning algorithms to predict carbon returns in China’s carbon trading market, and found that the random forest model had higher accuracy in predicting carbon returns in China than traditional econometric model. Gong et al. [13] chose extreme gradient boosting (XGBoost), light gradient boosting machine (LightGBM) and deep neural network (DNN) models to study how climate change impacts carbon futures returns. The findings indicate that the prediction accuracy markedly enhances when climate factors are incorporated into machine learning or deep learning models. Li et al. [14] used multivariate LSTM, multi-layer perceptron, support vector regression and recurrent neural network (RNN) for predicting Hubei emissions allowances (HBEA) and Guangdong emissions allowances (GDEA), and compare the prediction results with each other, which show that multivariate LSTM performs better for carbon price forecasting. Pan et al. [15] constructed an LSTM model that introduced particle swarm optimization to predict carbon prices and extracted effective keywords from Google search volume indices as inputs, which improved the prediction accuracy. Wang et al. [16] proposed a new hybrid prediction model based on a stacked ensemble learning algorithm. which integrates the echo state network, stacked autoencoder, support vector machine (SVM), and LSTM models as base learners, and a convolutional neural network (CNN) – bidirectional LSTM hybrid model as the meta-learner. This model effectively extracts time series features, and achieves high prediction accuracy and good generalisation ability.

AI models outperform traditional econometrics models in predicting nonlinear and non-stationary data. However carbon price data usually contain a large amount of noise and volatility, posing challenges for single models to handle complex, multilevel variations while ensuring predictive stability. For this reason, some scholars have proposed hybrid models and gradually improved them. Huang et al. [17] introduced a new decomposition-ensemble paradigm VMD-GARCH/LSTM-LSTM model, and found that the prediction errors of all decomposition-ensemble models are smaller than that of a single model, which confirms that decomposition-ensemble methods are effective in carbon price forecasting, and the study also shows that nonlinear integration algorithms can boost the prediction performance of multi-scale models. Wang et al. [18] developed a deep learning-based framework for forecasting carbon prices in Hubei province’s carbon trading pilot, and the study showed that principal component analysis and complete ensemble empirical mode decomposition with adaptive noise (CEEMDAN) methods significantly improve the accuracy of carbon price prediction.

In hybrid modeling, secondary decomposition has the obvious advantage of capturing the details that cannot be identified by primary decomposition and can better separate the noise from the useful signals. Sun and Huang [19] first decomposed the time series data into multiple intrinsic mode functions (IMFs) using empirical mode decomposition (EMD), and then applied variational mode decomposition (VMD) to the first intrinsic mode function (IMF1) for further decomposition. Their findings showed that model incorporating the quadratic decomposition algorithm achieved significantly higher prediction accuracy compared to those using undecomposed data or solely EMD methods. Compared to the EMD decomposition method, in order to extract the features of the signal more efficiently, Ozan [20] used multiple machine learning models optimized by CEEMDAN, VMD, and genetic algorithms, and found that the secondary decomposition markedly enhances model robustness and prediction accuracy by comparing the key performance metrics of the primary decomposition and secondary decomposition models. This outcome aligns with the findings of Sun and Huang. Zhou et al. [21] proposed a hybrid framework that combines CEEMDAN, sample entropy, VMD and LSTM. The study showed that the integrated VMD-LSTM method can effectively predict high-frequency time series extracted by CEEMDAN. In addition, through verification and comparison with different datasets, the study further proves that LSTM outperforms gated recurrent unit and DNN in carbon price forecasting. In order to further address the modal mixing issue in decomposition, Yang et al. [22] first performed a preliminary decomposition of carbon prices using ICEEMDAN, and then used multiscale fuzzy entropy to divide the decomposition results into high, medium, and low complexity components. After merging the high complexity components, they performed a second decomposition on the merged components using complete ensemble empirical mode decomposition. It is shown that ICEEMDAN surpasses other decomposition methods when dealing with data from Shenzhen and Hubei, and the secondary decomposition markedly diminishes data complexity, establishing a basis for subsequent precise predictions. Xu et al. [23] also employed an ICEEMDAN method, combined with a wavelet decomposition (WD) to perform a secondary decomposition of the original monthly runoff series. Subsequently, they utilised a seagull optimisation algorithm (SOA) optimised SVM to predict each component. This model framework effectively improved the prediction accuracy.

#### 1.1.2. Factors influencing the carbon market.

There is a wide range of literature that explores the factors affecting carbon prices, and these factors can be analyzed from both internal and external perspectives. In terms of internal factors, Sadefo and Njong [24] used a self-refinement to coarse reconstruction algorithm to decompose the subsequences and residuals extracted from the original carbon price into high-frequency, low-frequency, and trend components, aiming to deeply study the characteristics and basic laws of carbon prices in different frequency bands. Similarly, Zhu et al. [25] used improved random forest using particle swarm optimisation, extreme learning machine and LSTM to predict sequences of differing complexity, following the extraction of diverse features from the original carbon price series, and then combined and reconstructed them to better capture the characteristics of each component.

Although decomposition based on the historical data series of carbon price can effectively improve the prediction accuracy and generalization ability, it is limited by its disregard for external influences such as energy price and economic factors. To compensate for this deficiency, Li and Liu [26] classified external factors into structural and non-structural factors, and used Baidu search index as a non-structural data source. They highlighted that incorporating non-structural data can enhance the informational scope and consequently augment predictive accuracy. Similarly, Huang and He [27] refined the Baidu search index in feature selection by selecting the Baidu search index corresponding to low carbon economy, carbon trading, carbon emissions, carbon exchange rate, emission reduction, and carbon neutrality as unstructured influencing factors. Zhang et al. [28] also considered 20 factors related to carbon prices, including Baidu search index, when constructing a mixed model for forecasting carbon price. Finally, the Lasso method was used to extract variables strongly linked to carbon prices. The experimental results showed that the model performed well in terms of prediction accuracy and stability. Li et al. [29] selected text data from three sources: news reports, policy documents, and social commentary. They then integrated China’s daily time-series data on carbon emissions and the text data into a multimodal feature set using a convolutional neural network inceptionv2 and a text convolutional neural network optimised with an attention mechanism. This study comprehensively considered the factors influencing carbon emissions.

As for the structural factors, using external influences such as macroeconomics, energy prices, and international carbon markets as model inputs can substantially enhance the precision of carbon price forecasts [30–31]. Mao and Yu [32] used the SHAP method to analyze the key indicators of carbon price forecasting, and found that the carbon price was affected by the multiple influences of macroeconomic conditions, international carbon market prices, exchange rates, and domestic and international major energy prices. Zhang et al. [33] on the other hand, constructed a dynamic self-learning integrated forecasting framework, choosing Brent crude oil futures price and natural gas futures price as fuel and energy factors, and stoxx 600 index and goldman sachs commodity index (GSCI) as economic factors. The study demonstrates that the model achieves both high forecasting accuracy and stability. In addition, Hu and Cheng [34] selected the main external factors with the largest information coefficient based on the maximum information coefficient (MIC) method, revealing that the Guangdong carbon market price is heavily affected by the EUA price. Wang et al. [35] also selected features using the MIC, covering five aspects: fossil energy, macroeconomics, exchange rates, industrial development, and climate. The study showed that variables reflecting the overall economic situation, such as stock indices, exchange rates, and industrial development indices, have a significant positive contribution to the medium-term and long-term trend of carbon prices, while recent fossil fuel prices have a negative impact on the short-term trend of carbon prices due to supply and demand. It is worth noting that Wang et al. [36] also selected internal carbon market data such as opening price, maximum price and minimum price, and weather pollution such as air quality index (AQI) and PM2.5 as factors affecting the carbon price, and proposed an intelligent optimization decomposition and integration model based on multi-feature fusion, which achieves excellent prediction performance. Zheng et al. [37] also divided the factors affecting carbon emissions into three aspects. Among them, the carbon market aspect is reflected by the EU allowance futures carbon price (EUAP), EU allowances futures carbon trading volume and the Hubei carbon trading volume. The economic environment aspect is reflected by the dollar against the RMB exchange rate and CSl 300 index, etc., and the daily AQI of Wuhan City to reflect the natural environment. The study found that Brent crude oil futures settlement price and EUAP have a positive impact on carbon prices, while CSl 300 index has a negative impact on carbon prices.

### 1.2. Research gaps and contributions

Through the above literature review, it can be seen that although some scholars have achieved certain results in the research on carbon price forecasting, there are also shortcomings. First, most of the literature only focuses on the influence of conventional factors such as economy and energy, without taking domestic industry factors into account. Second, some studies do not consider whether the decomposition is sufficient in carbon price forecasting, and there are still problems of modal mixing and noise interference. Moreover, many studies still use the traditional hyperparameter optimization method for parameter adjustment, which cannot dynamically change the search range according to the actual results, and the search efficiency is low. Finally, some studies did not assign appropriate weights to different inputs in the final prediction, thus ignoring the contribution of important factors.

To this end, this paper proposes a hybrid model combining comprehensive feature screening, an optimized quadratic decomposition framework, and an Optuna-Attention-LSTM prediction method, providing the following contributions.

1. In this paper, we find the best XGBoost model parameters suitable for filling the missing values in each column through grid search and cross-validation. After finding the best combination of parameters, we use the trained XGBoost model to predict the missing values in the data. The model uses the known data to learn the pattern of missing values and fills in the unknown parts, thus supplementing the missing data in the three carbon markets.
2. In this paper, while considering external factors such as environmental and climate factors, energy factors, etc., we innovatively introduce domestic industry factors, adopt the feature selection method based on Boruta’s algorithm and LightGBM model, and synthesize it with Lasso’s regression feature selection method, and take the intersection of the filtered features and put it into the input of the final prediction, and at the same time take into account the nonlinear and linear feature.
3. The study combines ICEEMDAN and VMD to better extract the nonlinear features of carbon price, improves the quality of the reconstructed signal by noise reduction of the high-frequency component obtained by ICEEMDAN through SDAE, and introduces CPO algorithm to optimize the parameters in the process of VMD, which effectively improves the accuracy of decomposition.
4. In terms of model prediction, the optuna automated hyper-parameter optimization framework is innovatively introduced and combined with the attention mechanism to predict the prices of the three carbon markets through LSTM, improving prediction accuracy and stability.

## 2. Methodology

### 2.1. XGBoost

Iteratively filling in time series missing values based on machine learning models such as XGBoost can effectively reduce bias and improve the accuracy of data recovery in multiple scenarios [38]. As an algorithm based on gradient boosting decision trees, XGBoost incrementally enhances predictive accuracy by combining multiple weak learners.

To fill the target features containing missing values, XGBoost uses other features as input to train a model that can effectively predict these target features. The loss function is expressed as:


$$L(\theta )=\sum {\mathrm{\backslash nolimits}}_{i=1}^{n}l\left(y_{i},{\hat{y}}_{i}\right)+\sum {\mathrm{\backslash nolimits}}_{k=1}^{K}\Omega \left(f_{k}\right)$$ (1)

Where $\;y_{i}\;$ denotes the true value of the target feature containing the missing values, and $\;{\hat{y}}_{i}\;$ is the predicted value of the model, and the loss function $\;l(y_{i},{\hat{y}}_{i}\backslash )$ in this paper is in the form of negative mean square error, and $\;\Omega (f_{k}\backslash )$ is the regularization term, and the input feature set consists of other features through which the model predicts the target variable containing missing values.

To optimize the model, the hyperparameters are adjusted by a grid search $\;\theta =(\theta_{1},\theta_{2},\cdots ,\theta_{m}\backslash )$ to minimize the loss on the validation set. The optimization objective expression is given as:


$$\theta^{*}={argmin}_{\theta \in \Theta }\frac{1}{K}\sum {\mathrm{\backslash nolimits}}_{k=1}^{K}L_{val}^{\left(k\right)}(\theta )$$ (2)

Where $\;L_{val}^{\left(k\right)}(\theta$ denotes the loss of the validation set of the model in the kth-fold cross-validation, and *K* is the number of cross-validation folds. By traversing all possible combinations in the hyperparameter space  θ, the lattice search selects the optimal parameters that minimize the loss $\;\theta^{*}$, thus achieving accurate prediction of missing values.

### 2.2. Feature screening

#### 2.2.1. LightGBM based Boruta feature selection method.

In the Boruta framework, Random Forests are usually used to compute the importance of features, for example, Ghosh et al. [39] proposed an ensemble feature selection (EFS) method incorporating the Boruta and regularized random forest algorithms for filtering explanatory features. The effectiveness of combining the EFS process with advanced AI-based prediction models is well demonstrated. In this paper, we introduce the LightGBM model to be used as a base model in the Boruta algorithm to evaluate the relative significance of original and shadow features.

Let the closing price of the three carbon markets be the target variable, denoted as  y, where $\;y_{i}\;$ denotes the target value of the ith sample, and $\;x_{i}\;$ denotes the value of the feature over all samples. Boruta’s algorithm introduces shadow features, defined as:


$$X_{shadow}=\left[x_{s1},x_{s2},\ldots ,x_{sn}\right]$$ (3)

Among them, the shadow feature $\;x_{si}\;$ are obtained by randomly permuting the values of the original feature $\;x_{i}\;$ and do not contain any predictive information, so the shadow feature can be used as a benchmark for the uninformative features.

For a given feature $\;x_{i}$, its importance score $\;Imp(x_{i}\backslash )$ is defined as the cumulative split gain of the feature across all decision trees.


$$Imp\left(x_{i}\right)=\sum {\mathrm{\backslash nolimits}}_{j=1}^{T}\sum {\mathrm{\backslash nolimits}}_{k=1}^{N_{t_{j}}}\delta_{ijk}\cdot Gain\left(x_{i},t_{j},k\right)$$ (4)

Where  T  denotes the total number of decision trees in the LightGBM model, $N_{t_{j}}$ denotes the number of nodes in the jth tree, $\;\delta_{ijk}\;$ is an indicator function. When $\;x_{i}\;$ is used as the splitting feature at the kth node of the jth tree, $\;\delta_{ijk}=1;$ Otherwise $\;\delta_{ijk}=0$. Gain $\left(x_{i},t_{j},k\right)$ denotes the gain of the split at the kth node of the jth tree for the feature $\;x_{i}$.

By using the above formula, the importance scores of all shadow features are calculated and the maximum value is taken as the upper limit of the importance of the shadow feature, denoted as:


$${Imp}_{max}\left(X_{shadow}\right)=max\left(Imp\left(x_{s1}\right),Imp\left(x_{s2}\right),\ldots ,Imp\left(x_{sn}\right)\right)$$ (5)

Boruta’s algorithm performs multiple rounds of iterations, regenerating the shadow features and calculating the feature importance scores in each round, and then comparing them with the importance scores of the original features until all features are labeled as “confirmed” or “rejected”, or until the maximum number of preset iterations I is reached. The stability and reliability of the filtering results are ensured through multiple rounds of iterations. The final retained feature set is defined as:


$$X_{selected}=\{x_{i}|Imp\left(x_{i}\right)>{Imp}_{max}\left(X_{shadow}\right),\;i=1,\ldots ,n\}$$ (6)

#### 2.2.2. feature selection method based on Lasso regression.

The main reason for using linear models for feature selection in this paper is that financial data is noisy and nonlinear models may be too flexible and easy to extract irrelevant patterns from the noise, leading to poor feature selection [40].

Lasso regression controls the sparsity of features by adding a regularization term to the objective function of ordinary least squares regression. The optimization objective function for Lasso regression is defined as:


$$min_{\beta_{0},\beta }\left\{\frac{1}{2m}\sum {\mathrm{\backslash nolimits}}_{i=1}^{m}{\left(y_{i}-\beta_{0}-\sum {\mathrm{\backslash nolimits}}_{j=1}^{n}\beta_{j}x_{ij}\right)}^{2}+\lambda \sum {\mathrm{\backslash nolimits}}_{j=1}^{n}\left|\beta_{j}\right|\right\}$$ (7)

Where m is the number of samples, y is the target variable, $y_{i}$ is the target value of the ith sample, and $\;x_{ij}\;$ is the value of the jth feature of the ith sample. $\;\beta_{0}\;$ is the intercept term of the model.  β  is the regression coefficient corresponding to the feature.  λ  is the regularisation parameter, which controls the strength of $\;L_{1}\;$ regularisation.

In the optimization process, Lasso regression imposes sparsity constraints on some of the characteristic coefficients, resulting in a sparse vector of regression coefficients β. Ultimately, the model retains the set of features corresponding to non-zero coefficients as valid features. Let the solution of the feature coefficients be $\;\hat{\beta }$, then the final selected feature set is defined as:


$$X_{selected}=\{x_{j}|{\hat{\beta }}_{j}\neq 0,j=1,\ldots ,n\}$$ (8)

### 2.3. Secondary decomposition

#### 2.3.1. ICEEMDAN decomposition.

ICEEMDAN is further optimized on the basis of CEEMDAN algorithm, and its core idea is to add noise to the original signal and extract the IMF at each step. it can effectively capture the essence of the sequence and reduce the volatility of the data to improve the prediction accuracy [41], the steps of ICEEMDAN can be summarized as follows.

**Step 1.** Introduce a set of white noise signals for each sample $\;\in_{j}(t$ (where  j denotes the number of the noise sample, and ( j=1,2,…,M), and superimpose it with the original signal  x(t superimposed. Form the added noise signal.


$$x_{j}(t)=x(t)+\sigma \cdot \in_{j}(t)$$ (9)

Where  σ  is the noise amplitude.


$$IMF_{1}(t)=\frac{1}{M}\sum {\mathrm{\backslash nolimits}}_{j=1}^{M}IMF_{1,j}(t)$$ (10)

For each noise-added signal, EMD is used to extract, and calculate the average value of the first mode in all the added noise signals.

**Step 2.** For each subsequent modal function $\;IMF_{k}(t(k\geq 2)$, ICEEMDAN is extracted by recursion.


$$r_{k-1}(t)=x(t)-\sum {\mathrm{\backslash nolimits}}_{i=1}^{k-1}IMF_{i}(t)$$ (11)

The sum of the extracted modal functions is removed from the original signal  x(t to obtain the k-1th-order residual signal $\;r_{k-1}(t)$. Then, a new white noise $\;\in_{j}(t\;$ is added to each $\;r_{k-1}(t$ to form a new noise-added residual signal $\;r_{k-1,j}(t)$.


$$r_{k-1,j}(t)=r_{k-1}(t)+\sigma \cdot \in_{j}(t)$$ (12)

Then, for each $\;r_{k-1,j}(t)$, the kth-order modal function $\;IMF_{k,j}(t$ is extracted using EMD, and the kth-order modal mean of all the noise-added residuals is calculated to obtain the kth-order intrinsic modal function.


$$IMF_{k}(t)=\frac{1}{M}\sum {\mathrm{\backslash nolimits}}_{j=1}^{M}IMF_{k,j}(t)$$ (13)

**Step 3.** When the residual $\;r_{K}(t$ becomes a monotonic function or meets a preset energy threshold, the decomposition process is terminated and the final residual part no longer has a significant oscillatory component. At this point, the signal  x(t is represented as:


$$x(t)=\sum {\mathrm{\backslash nolimits}}_{k=1}^{K}IMF_{k}(t)+r_{K}(t)$$ (14)

Where  K  is the total number of modes obtained from the decomposition, and $\;r_{K}(t$ is the residual term.

#### 2.3.2. SDAE.

Stacked denoising autoencoder is a deep learning model for unsupervised learning. In this paper, stacked autoencoder is used for noise reduction of high frequency components after ICEEMDAN decomposition, the model consists of two layers of encoder and decoder, and the signal noise reduction is achieved by layer-by-layer dimensionality reduction and reconstruction.

**Step 1.** Input the matrix consisting of high frequency components IMF1 through IMF4 of size $\;X\in R^{2340\times 4}$*.*

Layer 1 encoder: input 2340 dimensional data, dimensionality reduction to 1170, then to 585. Where $\;W^{\left(1\right)}\in R^{1170}\;$ is the weight matrix, and $\;b^{\left(1\right)}\in R^{1170}\;$ is the bias vector  f(· is the activation function Tanh.


$$h^{\left(1\right)}=f(\begin{array}{c} W^{\left(1\right)}X+b^{\left(1\right)} \end{array})$$ (15)

Layer 1 decoder: reconstructs the 585-dimensional representation to a 2340-dimensional input size. Where $\;W^{(1top}\;$ is the transpose of the encoder’s weight matrix, and $\;c^{\left(1\right)}\;$ is the bias vector of the decoder, and  g(· is the activation function Tanh.


$${\hat{X}}^{\left(1\right)}=g\left(W^{(1)⊤}h^{\left(1\right)}+c^{\left(1\right)}\right)$$ (16)

Layer 2 encoder: receives the output of the layer 1 decoder and once again reduces the 2340 dimensional data to 1170, and finally to 585. Where $\;W^{\left(2\right)}\in R^{585\times 1170}\;$ is the weight matrix of the second layer encoder, and $\;b^{\left(2\right)}\;$ is the corresponding bias.


$$h^{\left(2\right)}=f\left(W^{\left(2\right)}{\hat{X}}^{\left(1\right)}+b^{\left(2\right)}\right)$$ (17)

Layer 2 decoder: restore the 585-dimensional representation to 2340 dimensions to generate the final denoised signal.


$${\hat{X}}^{\left(2\right)}=g\left(W^{(2)⊤}h^{\left(2\right)}+c^{\left(2\right)}\right)$$ (18)

**Step 2.** The input data  X  is reduced in dimension by two layer encoder and restored by two layer decoder to extract the signal characteristics and remove noise. The forward propagation process is as follows:


$${\hat{X}}^{\left(2\right)}=g\left(W^{(2)⊤}f\left(W^{\left(2\right)}g\left(W^{(1)⊤}f\left(W^{\left(1\right)}X+b^{\left(1\right)}\right)+c^{\left(1\right)}\right)+b^{\left(2\right)}\right)+c^{\left(2\right)}\right)$$ (19)

Where  X  denotes the input high frequency component containing noise, and $\;{\hat{X}}^{\left(2\right)}\;$ denotes the denoised signal after reconstruction.

**step3.** To realize the denoising effect, the model uses MSE as a loss function for calculating the error between the reconstructed signal and the target noise-free signal.


$$ℒ=\frac{1}{n}\sum {\mathrm{\backslash nolimits}}_{i=1}^{n}{\left(y_{i}-{\hat{x}}_{i}^{\left(2\right)}\right)}^{2}$$ (20)

Where $\;y_{i}\;$ is the ith sample value in the target noise-free signal, $\;{\hat{x}}_{i}^{\left(2\right)}\;$ is the ith sample value in the denoised reconstructed signal, and  n  is the number of samples. During training, the loss value  ℒ  is dynamically calculated for each iteration, and the Adam optimizer is used to minimize  ℒ  and gradually update the network parameters  W  and  b  to reduce the reconstruction error.

#### 2.3.3. VMD.

VMD is an adaptive signal processing method designed to decompose a complex signal into a set of IMFs with different center frequencies and narrowband characteristics, thereby effectively extracting features from the original time series [42]. Different from the traditional EMD, the VMD is based on the principle of bandwidth minimization and adopts a variational approach to decompose the signal, ensuring that the decomposed modes have good localization properties in the frequency domain.

The process of VMD is as follows:


$$\left\{ \begin{array}{c} min_{u_{k},\omega_{k}}\sum_{k}\left[\left(\delta (t)+\frac{j}{\pi t}\right)*u_{k}(t)\right]e^{-j\omega_{k}t}{∥}^{2}\\ s.t.\sum_{k}u_{k}(t)=f(t) \end{array}\right.\;$$ (21)

Where $\;u_{k}(t$ is the kth modal function, $\;\omega_{k}\;$ is the center frequency of the modal function $\;u_{k}(t)$, and $\;\delta (t)+\frac{j}{\pi t}\;$ is the composite function of the Dirac function and the Hilbert transform, which is used to obtain the analytic signal of the signal,  *  indicates the convolution operation, and $\;e^{-j\omega_{k}t}\;$ is the frequency shift factor, which shifts the modal function to near the center frequency $\;\omega_{k}$.


$$ℒ\left(u_{k},\omega_{k},\lambda \right)=\alpha \sum {\mathrm{\backslash nolimits}}_{k=1}^{K}∥\partial_{t}\left[\left(\delta (t)+j\frac{1}{\pi t}\right)*u_{k}(t)\right]e^{-j\omega_{k}t}{∥}_{2}^{2}$$

$$+∥f(t)-\sum {\mathrm{\backslash nolimits}}_{k=1}^{K}u_{k}(t){∥}_{2}^{2}+\left\langle \lambda (t),f(t)-\sum {\mathrm{\backslash nolimits}}_{k=1}^{K}u_{k}(t)\right\rangle$$ (22)

Where  α  is a balance parameter that controls the trade-off between bandwidth minimization and reconstruction accuracy, and  λ(t is the Lagrange multiplier for imposing constraints on the decomposition.

VMD employs the alternating direction multiplier method (ADMM) to address the above optimization problem. ADMM gradually approaches the global optimal solution by separately optimizing the modal function $\;u_{k}\;$ and the center frequency $\;\omega_{k}$, as well as the Lagrange multiplier  λ, in each iteration. Fixing $\;\omega_{k}\;$ and  λ, the update formula for the modal function $\;u_{k}\;$ is solved by minimizing the partial derivative of the Lagrangian function with respect to each $\;u_{k}$.


$$u_{k}^{\left(n+1\right)}(t)=IFT\left\{\frac{\hat{f}(\omega )-\sum_{i\neq k}{\hat{u}}_{i}^{\left(n\right)}(\omega )+\frac{\lambda (\omega )}{2}}{1+2\alpha {\left(\omega -\omega_{k}^{\left(n\right)}\right)}^{2}}\right\}$$ (23)

Where  IFT  denotes the inverse Fourier transform and $\;{\hat{u}}_{k}(\omega$ is the frequency domain representation of the modal function $\;u_{k}$.

Then, fixing $\;u_{k}\;$ and  λ, minimizing the partial derivative of the Lagrangian function with respect to $\;\omega_{k}$, we obtain an updated formula for the central frequency.


$$\omega_{k}^{\left(n+1\right)}=\frac{\int_{0}^{\infty }\omega {\left|{\hat{u}}_{k}^{\left(n+1\right)}(\omega )\right|}^{2}d\omega }{\int_{0}^{\infty }{\left|{\hat{u}}_{k}^{\left(n+1\right)}(\omega )\right|}^{2}d\omega }$$ (24)

Finally the Lagrange multipliers are updated to progressively satisfy the constraints. Where  τ  is the step parameter, which is used to control the speed of convergence of the Lagrange multipliers.


$$\lambda^{\left(n+1\right)}(t)=\lambda^{\left(n\right)}(t)+\tau \left(f(t)-\sum {\mathrm{\backslash nolimits}}_{k=1}^{K}u_{k}^{\left(n+1\right)}(t)\right)$$ (25)

The VMD algorithm, by iterating the above steps repeatedly, makes the modal function $\;u_{k}\;$ and center frequency $\;\omega_{k}\;$ gradually converge to a steady state, thus obtaining a modal decomposition result that satisfies the bandwidth minimization condition.

#### 2.3.4. CPO.

CPO is a biomimetic optimization algorithm that simulates the group defense behavior of the crown porcupines to achieve a global optimal solution search in the balance of exploration and development. The defense mechanism of the crown porcupines includes vision, sound, smell and physical attacks, among which vision and sound correspond to the exploration operator, and smell and physical attacks correspond to the development operator. In addition, CPO also introduces the cyclic population reduction technique to preserve population diversity and enhance the convergence speed [43].

**Step1.** In the initialization phase, the positions of the individuals are randomly distributed within the given upper and lower bounds, and the position of the ith individual in the population is initialized as follows:


$$X_{ij}={lb}_{j}+rand\times \left({ub}_{j}-{lb}_{j}\right)$$ (26)

Where $\;{lb}_{j}\;$ and $\;{\text{ub}}_{j}\;$ are the lower and upper bounds of the jth dimension respectively, and rand is a random number in the range [0,1].

**Step2.** In each iteration, the CPO updates the position of an individual through two mechanisms: exploration and exploitation.

The exploration phase simulates the defensive extension behavior of the crown porcupine with the individual position update equation.


$$X_{i}=X_{i}+\alpha \cdot r_{1}\cdot \left(X_{\text{rand}}-X_{\text{mean}}\right)$$ (27)

Where  α  is a coefficient that controls the exploration range, $\;r_{1}\;$ is a random number used to increase the diversity of individuals, $\;X_{rand}\;$ is a random individual in the population, and $\;X_{mean}\;$ is the average of the current population position.

The development phase corresponds to the clustering behavior between individuals, and its position update formula is:


$$X_{i}=X_{\text{best}}+\beta \cdot r_{2}\cdot \left(X_{\text{best}}-X_{i}\right)$$ (28)

Where $\;X_{best}\;$ is the individual with optimal fitness in the current population, and  β  is the control parameter for the convergence step size, and $\;r_{2}\;$ is a [0,1] random number.

**Step 3.** After updating the position, if the individual exceeds the boundary, the boundary reflection mechanism is used to keep it within the reasonable range.


$$X_{ij}=\left\{ \begin{array}{cc} {lb}_{j}+rand\times \left({ub}_{j}-{lb}_{j}\right) & ifX_{ij}<{lb}_{j}\\ {ub}_{j}-rand\times \left({ub}_{j}-{lb}_{j}\right) & ifX_{ij}>{ub}_{j} \end{array}\right.\;$$ (29)

**Step 4.** At the end of each iteration, the fitness of every individual in the population is assessed, and the prevailing optimal solution is retained as the global optimal solution of the population. If the new solution surpasses the global optimal solution, the global optimal solution is updated.

### 2.4. Optuna-Attention-LSTM predictive hybrid modeling

#### 2.4.1. LSTM.

**Fig 1:**
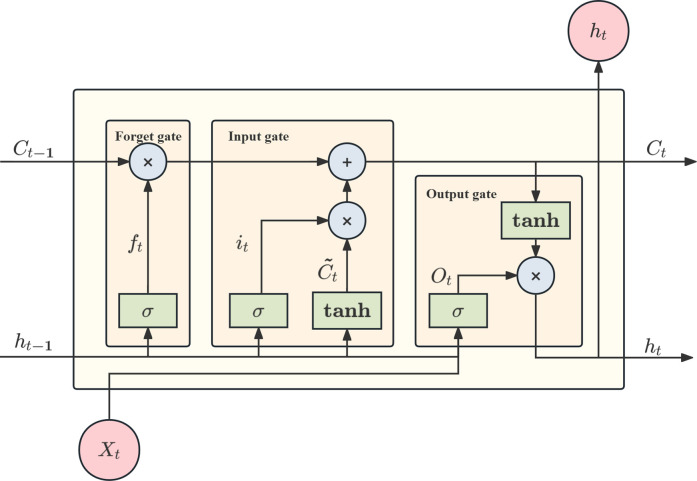
Structure of LSTM.

LSTM is an improved model of RNN. Compared with traditional RNN, LSTM significantly enhances the learning ability of long-term dependencies through a structured network design, and shows stronger predictive accuracy in tasks involving long sequence data [44]. The LSTM effectively controls the flow of information at each time step by introducing a structure with input gates, forget gates and output gates, as shown in Fig 1, enabling the model to retain important information in long sequence data and suppress cumulative errors. The computational process of the LSTM unit consists of four steps:

(1) Oblivion gate: based on the hidden state $\;h_{t-1}\;$ of the previous time step and the current input $\;x_{t}$, the output $\;f_{t}\;$ of the forgetful gate is calculated to determine the part of the previous momentary cell state $\;c_{t-1}\;$ that needs to be forgotten.


$$f_{t}=\sigma \left(W_{f}\cdot \left[h_{t-1},x_{t}\right]+b_{f}\right)$$ (30)

(2) Input gate: determines the importance of the current input $\;x_{t}$*.* The input gate $\;i_{t}\;$ and the candidate memory state $\;{\tilde{c}}_{t}\;$ are calculated as follows:


$$i_{t}=\sigma \left(W_{i}\cdot \left[h_{t-1},x_{t}\right]+b_{i}\right)$$ (31)

$${\tilde{c}}_{t}=tanh\left(W_{c}\cdot \left[h_{t-1},x_{t}\right]+b_{c}\right)$$ (32)

(3) Updating the cell state: combining the outputs of the forgetting gate and the input gate, the cell state $\;c_{t}\;$ at the current moment is updated.


$$c_{t}=f_{t}⊙c_{t-1}+i_{t}⊙{\tilde{c}}_{t}$$ (33)

(4) Output gate: calculate the output gate $\;o_{t}$, and based on the current cell state $\;c_{t}$ update the hidden state $\;h_{t}$. Where  σ  denotes the sigmoid activation function, and  ⊙  denotes the element level multiplication.


$$o_{t}=\sigma \left(W_{o}\cdot \left[h_{t-1},x_{t}\right]+b_{o}\right)$$ (34)

$$h_{t}=o_{t}⊙tanh\left(c_{t}\right)$$ (35)

#### 2.4.2. Attention mechanism.

The Attention mechanism allocates weights to each component of the input sequence, allowing the model to concentrate on critical elements and enhancing its capacity to capture temporal or spatial relationships. The mechanism obtains weighted output by calculating the similarity between the vector  Q, vector  K, and vector  V. The calculation formula is:


$$Attention(Q,K,V)=softmax\left(\frac{QK^{T}}{\sqrt{d_{k}}}\right)V$$ (36)

Where $\;Q\in R^{n\times d_{q}}\;$ is the query matrix, and $\;K\in R^{n\times d_{k}}\;$ is the key matrix, and $\;V{\in R}^{n\times d_{v}}\;$ the value matrix, and $\;d_{k}\;$ denotes the dimension of the key vector, which is used as a scaling factor to stabilize the range of values.

#### 2.4.3. Optuna.

Optuna is an automated hyperparameter optimization framework. Its “define-by-run” feature allows users to flexibly define the hyperparameter space and search for the optimal hyperparameter combination in a parallelized manner. In addition, the framework enables users to query and document the hyperparameter values throughout the optimization process [45].

Optuna defines the hyperparameter optimization problem as the minimization of an objective function. In this framework, the goal is to identify a set of hyperparameters  θ  that minimizes the objective function  f(θ).


$$min_{\theta }f(\theta )$$ (37)

Where  f(θ denotes the model performance metric determined by the set of hyperparameters  θ, and  θ  denotes the combination of hyperparameters sampled from the defined parameter space. Optuna uses the Tree-structured Parzen Estimator algorithm to quickly converge to the optimal solution by dynamically adjusting the posterior probability distribution to guide the sampling of the next set of hyperparameters.

## 3. The proposed approach

This paper proposes a hybrid model that integrates comprehensive feature screening, an optimized quadratic decomposition framework, and the Optuna-Attention-LSTM prediction method.

**Fig 2:**
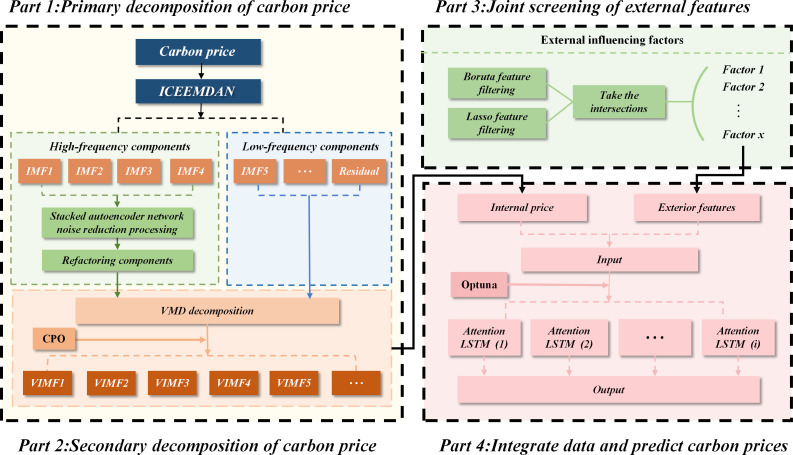
Flowchart of the overall process of the hybrid model.

(1) In this paper, the ICEEMDAN model is first employed to decompose the carbon price time series into IMF sub-series of different frequencies, until decomposition to the residual. Furthermore, the g test reconstruction algorithm is used to differentiate between high and low frequencies in the IMF. To mitigate noise interference in high-frequency components, the SDAE method is employed for denoising. Subsequently, the denoised high-frequency components are combined with the low-frequency components following primary decomposition to reconstruct the signal.
(2) Then the reconstructed signal is decomposed quadratically by the VMD method, and the CPO algorithm is introduced for parameter optimization by substituting the data fitting penalty alpha, the number of modes K, and the bandwidth penalty tau into the adaptive function, and the optimal solution is continuously iteratively updated to control the VMD, after that, VIMF1 and other components are obtained.
(3) The feature engineering is mainly segmented into two phases: the first phase first summarizes the external factors affecting the carbon price based on previous research and adds several domestic industry factors, then screens different factors in each type by Boruta; the second phase uses Lasso regression to re-screen the features, and finally, by taking the intersection of the features screened in the two stages, the intersection of the two stages is taken to determine the model’s final input features.
(4) Finally, the decomposed VIMF sequence and the screened external factors are predicted by LSTM model. Attention mechanism is introduced to allocate varying weights to different inputs during prediction, and optuna is used for hyperparameter optimization. Then, the predicted values of all components are added to derive the final prediction result. The overall model process is shown in Fig 2.

## 4. Empirical analysis

### 4.1. Data description

The data used in this study mainly includes data related to carbon prices and external factors influencing them. Data sources include the Choice financial terminal, the Wind database, and http://www.tanjiaoyi.com.

#### 4.1.1. Closing price data.

**Fig 3:**
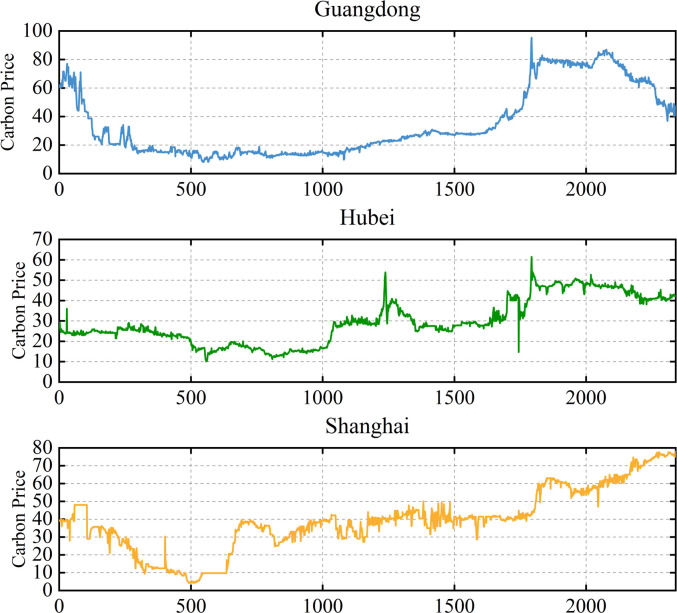
Trends of the original price series of the three carbon markets.

The study used daily carbon prices in Hubei, Guangdong, and Shanghai from April 4, 2014 to September 30, 2024 as the experimental data, and the first 85% of the dataset was chosen as the training set and the last 15% as the test set. Fig 3 shows the trend of the original price series across the three carbon markets.

#### 4.1.2. Influence factor data.

The model in this paper takes into consideration the factors influencing the carbon price, including structural and non-structural factors.

1. Structural factors

The structural factors covered in this paper include environmental and climatic factors, energy factors, carbon market factors, exchange rate and monetary factors, domestic industry factors, and economic factors.

(1) Environmental and climatic factors

In this paper, daily AQI, Daily maximum temperature and Daily minimum temperature are used to reflect the impact of environment and climate on carbon price. In particular, the relevant data of Guangdong province and Hubei province are replaced by AQI, maximum air temperature and minimum air temperature data of Guangzhou city and Wuhan city, respectively.

(2) Energy factors

In this paper, the Brent crude oil futures settlement price, Daqing crude oil spot price, Thermal coal futures closing price, China coking coal composite index, and NYMEX natural gas closing price are selected to represent the impact of energy on the carbon price, in which the Thermal coal futures closing price comes from Zhengzhou commodity exchange.

(3) Carbon market factors

In this paper, EUA futures closing price, EUA futures volume, GDEA volume, HBEA volume, and Shanghai emission allowance (SHEA) volume are chosen to represent the EU carbon market and the impact of internal factors of the carbon market on carbon price.

(4) Exchange rates and monetary factors

In the paper, the USD to CNY exchange rate, the USD to EUR exchange rate, and the RMB index are chosen to reflect the influence of exchange rate and monetary factors on carbon prices in the three markets.

(5) Domestic industry factors

In this paper, the Chemicals index, Textile index, Steel index, Energy index, Agricultural and sideline products index, Rubber and plastic index, Non-ferrous metals index, and Commodity price index are selected to reflect the impact of domestic industries on the carbon prices of the three markets.

(6) Economic factors

In the paper, NASDAQ index, S&P 500 index, SSE composite index volume, SSE composite index closing price, CSI 300 index volume, CSI 300 index closing price, SHFE: Gold futures closing price, SHFE: Silver futures closing price, and BDI index are selected to react to the influence of economic factors on the price of carbon market.

1. Unstructural factors

Unstructured indicators can capture market sentiment, public attention and behavioral changes that are difficult to be reflected in traditional structured data, making the information more comprehensive. This study uses Baidu search index as unstructured data, collecting daily search volumes for relevant keywords in Guangdong, Hubei, and Shanghai. Keywords include carbon, low carbon, low carbon economy, energy conservation, emission reduction, carbon sink, carbon emission, carbon trading, carbon emission trading, carbon footprint, carbon neutrality. Finally, the search volume of all keywords in the region is summed as the unstructured data for the region. This study selects data from the same period to match all variables.

#### 4.1.3. Data preprocessing.

This paper first employs the XGBoost method to impute all missing values, using the complete features as input. By learning the complex nonlinear relationships among features, it effectively predicts missing data and significantly improves the accuracy of imputation. Subsequently, to eliminate the impact of different feature dimensions, normalisation is applied to scale all data to the [0, 1] range, standardising the feature scale. Next, to construct training samples suitable for predicting carbon emission allowance closing prices, a sliding window length of 10 is set, corresponding to two weeks of trading days. Finally, the processed data set is divided into a training set comprising the first 85% and a test set comprising the remaining 15% for model training and performance evaluation. During parameter tuning, we used a time series cross-validation method to divide the training set into five folds in chronological order. In each fold, the training set was always earlier than the validation set to match the actual scenario of time series modelling and avoid information leakage.

### 4.2. Measuring indicators

In order to assess the performance of the prediction model in predicting the carbon prices in Guangdong, Hubei and Shanghai, this paper calculates the following four different statistical metrics: MSE, RMSE, MAE and MAPE as shown in Table 1, where n is the total number of samples and $y_{i}$ is the ith sample of the true value, and ${\hat{y}}_{i}$ is the ith sample of the predicted value. Lower values of these metrics indicate reduced errors and better prediction accuracy.

**Table 1:** Formulaic table of measurement indicators.

Norm	Formula
MSE	$\begin{array}{c} \frac{1}{n}\sum_{i=1}^{n}{\left(y_{i}-{\hat{y}}_{i}\right)}^{2} \end{array}$
RMSE	$\begin{array}{c} \sqrt{\frac{1}{n}\sum_{i=1}^{n}{\left(y_{i}-{\hat{y}}_{i}\right)}^{2}} \end{array}$
MAE	$\begin{array}{c} \frac{1}{n}\sum_{i=1}^{n}\left|y_{i}-{\hat{y}}_{i}\right| \end{array}$
MAPE	$\begin{array}{c} \frac{1}{n}\sum_{i=1}^{n}\left|\frac{y_{i}-{\hat{y}}_{i}}{y_{i}}\right|\times 100\\ \% \end{array}$

### 4.3. Secondary decomposition process

#### 4.3.1. ICEEMDAN decomposition and denoising.

**Fig 4:**
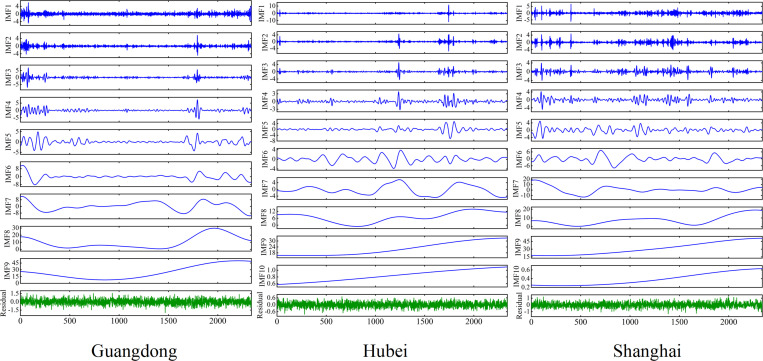
Primary decomposition of carbon price ICEEMDAN.

This study employs the ICEEMDAN method to decompose the original carbon price series from three carbon markets into modal components of differing frequencies. As illustrated in Fig 4, the IMF1 sequence has the highest frequency, and the subsequent IMF frequency gradually decreases, and the fluctuations gradually level off. From the figure, it can be seen that the trend of the low-frequency components and the trend of the original series as a whole are roughly consistent, and the trend of the low-frequency components is more gentle.

Next, the g test is applied to differentiate the high-frequency and low-frequency components in the subsequence to improve the plausibility of sequence reconstruction. Table 2 shows the g values of the sub-sequences after the ICEEMDAN decomposition of the carbon prices in the three markets. For the Guangdong carbon market, it can be seen that the g-values of IMF1 to IMF3 are low, and the percentages are all below 10%, which are typical high-frequency components. While IMF4 belongs to a turning point and no longer behaves as a typical high-frequency component, the percentage is significantly higher than that of IMF3 but still lower than that of IMF6, which is a transition component and may contain part of the high-frequency signals, so it is also considered within the high-frequency component, and the rest are low-frequency components. For the Hubei carbon market, it can be seen that the g-values from IMF1 to IMF3 are lower, and IMF4 belongs to a turning point, which is a transition component, so it is also considered in the high-frequency component, and the rest are low-frequency components. For Shanghai carbon market, it can be seen that the g-values from IMF1 to IMF4 are low, and the percentages are all below 3%, then IMF1, IMF2, IMF3 and IMF4 are high-frequency components, and the rest are low-frequency components.

**Table 2:** High-frequency and low-frequency IMF components in the three carbon markets.

Carbon market	Intrinsic modal functions	G-value	Percentage	Frequency
Guangdong carbon market	IMF1	0.201	8.721	high
	IMF2	0.142	6.161	high
	IMF3	−0.079	3.446	high
	IMF4	−0.828	35.885	high
	IMF5	−0.437	18.959	low
	IMF6	2.949	127.896	low
	IMF7	3.350	145.259	low
	IMF8	4.956	214.913	low
	IMF9	7.343	318.420	low
Hubei carbon market	IMF1	−0.231	10.213	high
	IMF2	0.262	11.566	high
	IMF3	0.197	8.714	high
	IMF4	0.350	15.472	high
	IMF5	0.084	3.696	low
	IMF6	0.214	9.466	low
	IMF7	−0.303	13.390	low
	IMF8	3.770	166.650	low
	IMF9	6.407	283.243	low
	IMF10	0.238	10.518	low
Shanghai carbon market	IMF1	0.015	0.650	high
	IMF2	0.063	2.780	high
	IMF3	−0.027	1.203	high
	IMF4	−0.009	0.391	high
	IMF5	−0.403	17.812	low
	IMF6	−0.682	30.150	low
	IMF7	5.124	226.502	low
	IMF8	1.949	86.177	low
	IMF9	5.181	229.011	low
	IMF10	0.073	3.232	low

Next, a stacked auto-encoder is used to perform noise reduction on the high-frequency components IMF1 to IMF4, after which the noise-reduced IMF1, IMF2, IMF3, IMF4, and the low-frequency components are summed up to obtain the reconstructed signals of the primary decomposition.

#### 4.3.2. Optimized VMD decomposition.

**Fig 5:**
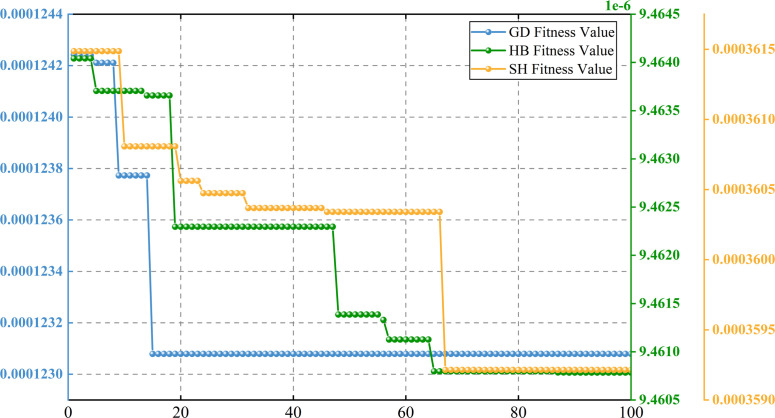
Convergence curve of CPO optimization algorithm for carbon market.

In order to effectively eliminate mode aliasing. The VMD decomposition is started after the primary decomposition, and CPO is introduced to optimize the alpha, tau, and K parameters in the decomposition process to determine the best decomposition parameters. Fig 5 shows the convergence curves of the three carbon markets, which all show the effectiveness of the CPO algorithm in application. The detailed parameter settings for CPO algorithm are shown in Table 3.

**Table 3:** Specific details of CPO optimisation of VMD decomposition.

Carbon market	Parameter category	Parameter range	Number of iterations	Final parameters	Fitness value
Guangdong	alpha	[1000,3000]	100	1785.952	1.231e-4
	K	[6,10]	100	9	1.231e-4
Hubei	alpha	[1000,3000]	100	1304.636	9.461e-6
	K	[6,10]	100	8	9.461e-6
Shanghai	alpha	[1000,3000]	100	1795.785	3.592e-4
	K	[6,10]	100	9	3.592e-4

**Fig 6:**
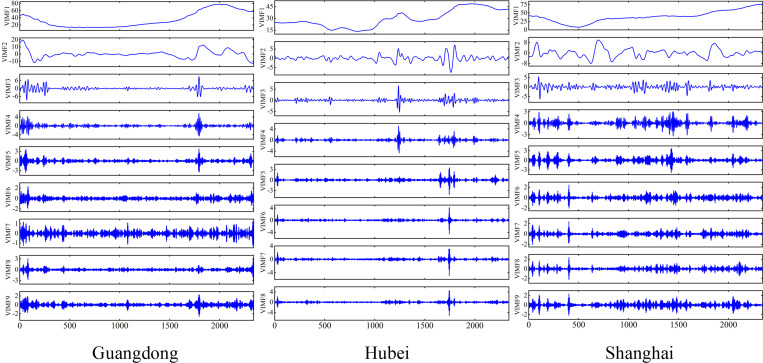
VMD decomposition results for three carbon markets.

Finally, the optimal K value of Guangdong carbon market and Shanghai carbon market is determined to be 9, which is decomposed into 9 modal components, and the optimal K value of Hubei carbon market is 8, which is decomposed into 8 modal components. Fig 6 specifically shows the time domain waveforms of different modal components.The sensitivity analysis of VMD parameters for the three carbon markets is detailed in Tables 4–5, and Table 6.

**Table 4:** Parameter sensitivity analysis of VMD decomposition in the Guangdong carbon market.

Carbon market	Modal component	Bandwidth penalty factor	Fitness value
Guangdong	K = 6	alpha = 1000	0.075
		alpha = 1500	0.118
		alpha = 2000	0.313
		alpha = 2500	0.507
		alpha = 3000	0.564
	K = 7	alpha = 1000	0.012
		alpha = 1500	0.208
		alpha = 2000	0.061
		alpha = 2500	0.169
		alpha = 3000	0.478
	K = 8	alpha = 1000	0.009
		alpha = 1500	0.041
		alpha = 2000	0.144
		alpha = 2500	0.251
		alpha = 3000	0.314
	K = 9	alpha = 1000	1.242e-4
		alpha = 1500	0.013
		alpha = 2000	0.047
		alpha = 2500	0.017
		alpha = 3000	0.142
	K = 10	alpha = 1000	1.242e-4
		alpha = 1500	0.001
		alpha = 2000	0.041
		alpha = 2500	0.074
		alpha = 3000	0.086

**Table 5:** Parameter sensitivity analysis of VMD decomposition in the Hubei carbon market.

Carbon market	Modal component	Bandwidth penalty factor	Fitness value
Hubei	K = 6	alpha = 1000	0.032
		alpha = 1500	0.223
		alpha = 2000	0.149
		alpha = 2500	0.105
		alpha = 3000	0.069
	K = 7	alpha = 1000	0.001
		alpha = 1500	0.037
		alpha = 2000	0.153
		alpha = 2500	0.067
		alpha = 3000	0.132
	K = 8	alpha = 1000	9.464e-6
		alpha = 1500	0.006
		alpha = 2000	0.043
		alpha = 2500	0.189
		alpha = 3000	0.066
	K = 9	alpha = 1000	9.464e-6
		alpha = 1500	9.693e-6
		alpha = 2000	0.006
		alpha = 2500	0.035
		alpha = 3000	0.059
	K = 10	alpha = 1000	9.464e-6
		alpha = 1500	9.464e-6
		alpha = 2000	0.005
		alpha = 2500	0.009
		alpha = 3000	0.019

**Table 6:** Parameter sensitivity analysis of VMD decomposition in the Shanghai carbon market.

Carbon market	Modal component	Bandwidth penalty factor	Fitness value
Shanghai	K = 6	alpha = 1000	0.061
		alpha = 1500	0.198
		alpha = 2000	0.304
		alpha = 2500	0.140
		alpha = 3000	0.554
	K = 7	alpha = 1000	0.007
		alpha = 1500	0.024
		alpha = 2000	0.242
		alpha = 2500	0.315
		alpha = 3000	0.730
	K = 8	alpha = 1000	0.002
		alpha = 1500	0.018
		alpha = 2000	0.190
		alpha = 2500	0.140
		alpha = 3000	0.077
	K = 9	alpha = 1000	3.616e-4
		alpha = 1500	0.003
		alpha = 2000	0.013
		alpha = 2500	0.014
		alpha = 3000	0.043
	K = 10	alpha = 1000	3.616e-4
		alpha = 1500	8.664e-4
		alpha = 2000	9.709e-3
		alpha = 2500	0.102
		alpha = 3000	0.119

Through the VMD decomposition, the raw signal of Hubei carbon market is decomposed into VIMF1 to VIMF8, where VIMF1 and VIMF2 correspond to the low-frequency trend of the signal, VIMF3 to VIMF5 capture the mid-frequency dynamics of the signal, and VIMF6 to VIMF8 separate the high-frequency oscillations and noise in the signal. The original signals of Guangdong carbon market and Shanghai carbon market are decomposed into VIMF1 to VIMF9, where VIMF1 to VIMF3 show the low-frequency trend components, VIMF3 to VIMF6 gradually capture the mid-frequency characteristics, and VIMF7 to VIMF9 mainly reflect the high-frequency components. Overall, the optimized VMD decomposition achieves an accurate separation of the signal in the time and frequency domains.

### 4.4. Screening of external factors

#### 4.4.1. Comprehensive feature screening.

**Fig 7:**
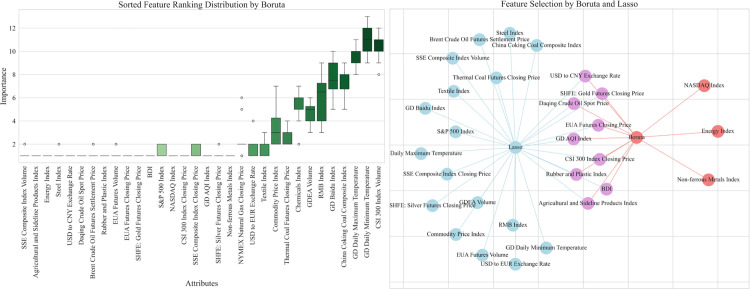
Comprehensive characteristic screening results of Guangdong carbon market.

By selecting the top and stable elements, the Boruta algorithm identified 13 key factors in the Guangdong carbon market, including the CSI 300 index closing price, NASDAQ index, etc. By Lasso regression method, SSE composite index volume, SSE composite index closing price and other factors were screened in Guangdong carbon market. Finally, through the comprehensive feature screening by Boruta algorithm and Lasso regression, we determined the intersection of the selected feature variables of the two methods as shown in Fig 7. These commonly contained features are finally used as inputs to the forecasting model, including USD to CNY exchange rate, Daqing crude oil spot price, etc.

**Fig 8:**
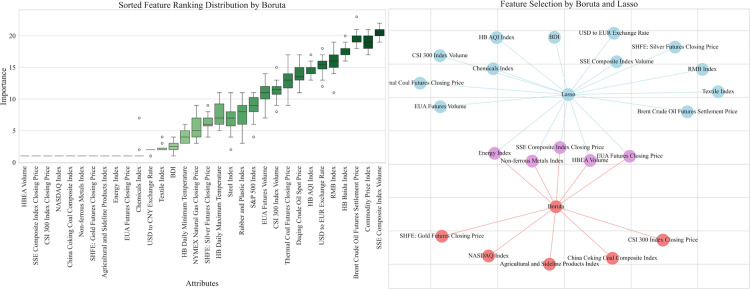
Comprehensive characteristic screening results of Hubei carbon market.

**Fig 9:**
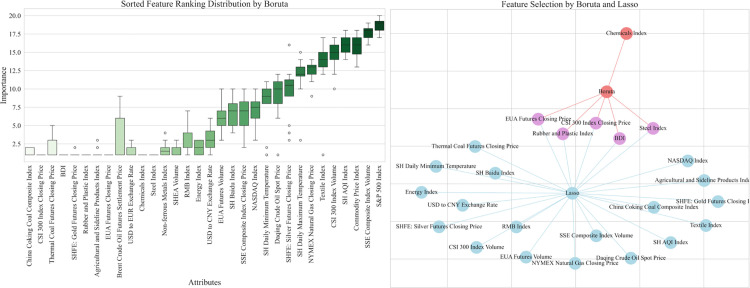
Comprehensive characteristic screening results of Shanghai carbon market.

Hubei carbon market and Shanghai carbon market also screened the final input external factors through the above process as shown in Figs 8 and 9. The results of the final screening for the three carbon markets are detailed in Table 7.

**Table 7:** Screening results for the characteristics of the three carbon markets.

Carbon market	Typology	Factor
Guangdong carbon market	Environmental and climatic factors	GD AQI Index
	Economic factor	CSl 300 Index Closing Price
		SHFE: Gold Futures Closing Price
		BDI
	Exchange rates and monetary factors	USD to CNY Exchange Rate
	Domestic industry factors	Rubber and Plastic Index
		Agriculturat and Sideline Products Index
	Carbon market factors	EUA Futures Closing Price
	Energy factors	Daqing Crude Oil Spot Price
Hubei carbon market	Economic factor	SSE Composite index Closing Price
	Domestic industry factors	Energy Index
		Non-ferrous Metals Index
	Carbon market factors	EUA Futures Closing Price
		HBEA Volume
Shanghai carbon market	Economic factor	CSl 300 Index Closing Price
		BDI
	Domestic industry factors	Rubber and Plastic Index
		Steel Index
	Carbon market factors	EUA Futures Closing Price

### 4.5. Forecasting process

**Fig 10:**
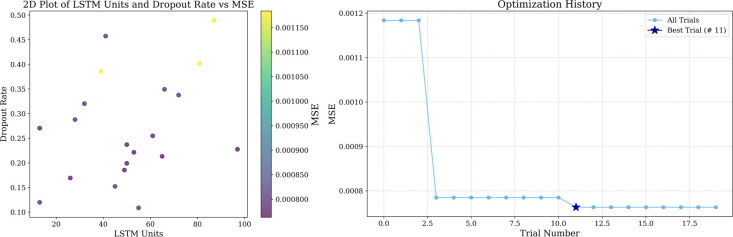
Optuna hyperparameter tuning results in Guangdong market.

**Fig 11:**
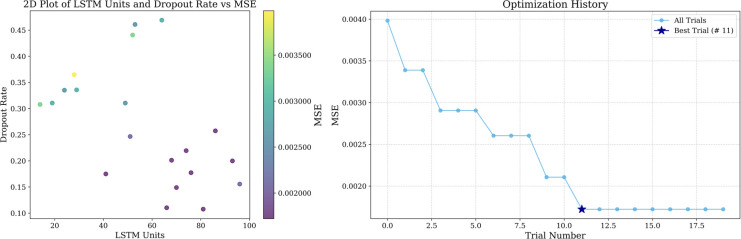
Optuna hyperparameter tuning results in Hubei market.

**Fig 12:**
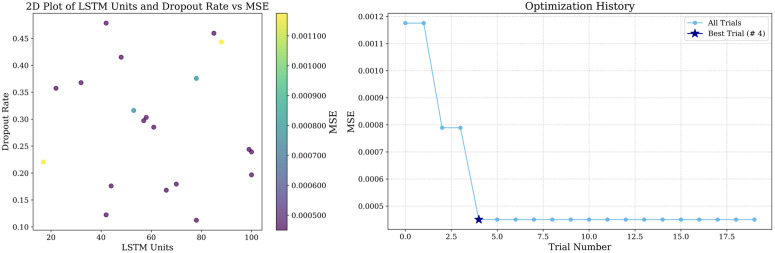
Optuna hyperparameter tuning results in Shanghai market.

In prediction if the selected time step is too short, it may not be able to cover enough historical information, and if the time step is too long more noise may be introduced, so in this paper, we set up to predict the value of the next time point with the data of the previous 10 time steps and use a two-layer LSTM, the first layer of the LSTM is used to generate the hidden state of the time step, and the second layer is used to further extract the features, and we also add an attention mechanism to pay attention to the important part of the LSTM output sequence. LSTM units increase will enhance the model complexity, while the dropout rate can prevent the model complexity from being too high leading to overfitting, in order to balance the model’s learning ability and generalization ability, this paper adopts optuna to tune LSTM units and dropout rate, and set to carry out 20 experiments. The tuning results are shown in Figs 10, 11 and 12, and the specific parameters listed in Table 8.

**Table 8:** Optuna hyperparameter optimization results.

Carbon market	Parameter category	Parameter range	Number of iterations	Final parameters	MSE
Guangdong	LSTM units	[10,100]	20	61	7.631e-4
	dropout rate	[0.1,0.5]	20	0.254	7.631e-4
Hubei	LSTM units	[10,100]	20	66	1.721e-3
	dropout rate	[0.1,0.5]	20	0.110	1.721e-3
Shanghai	LSTM units	[10,100]	20	48	4.500e-4
	dropout rate	[0.1,0.5]	20	0.415	4.500e-4

**Fig 13:**
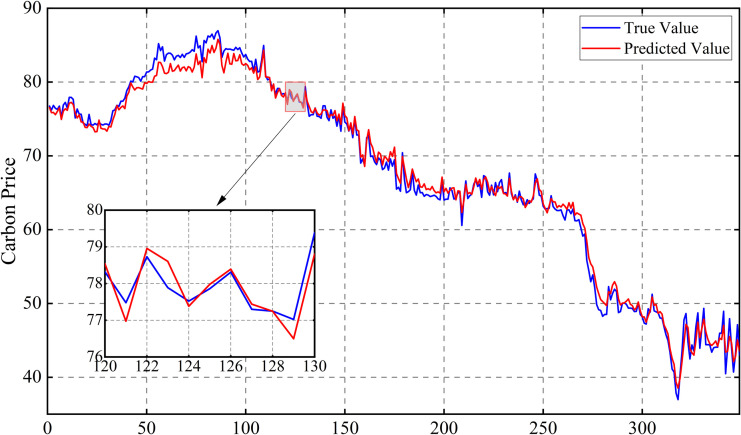
Results of carbon price forecasts for the Guangdong market.

**Fig 14:**
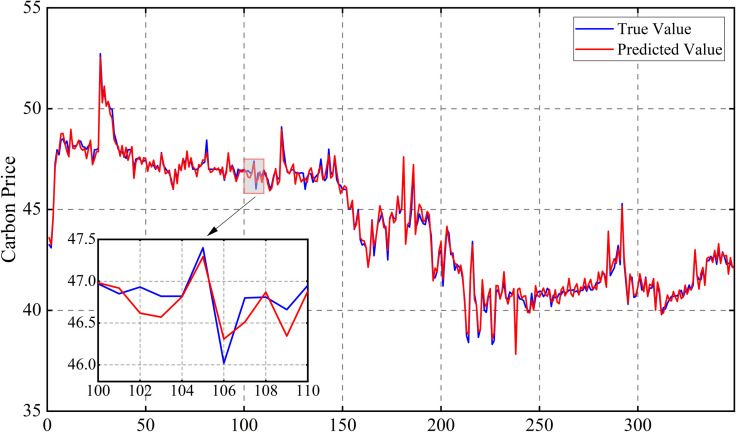
Results of carbon price forecasts for the Hubei market.

**Fig 15:**
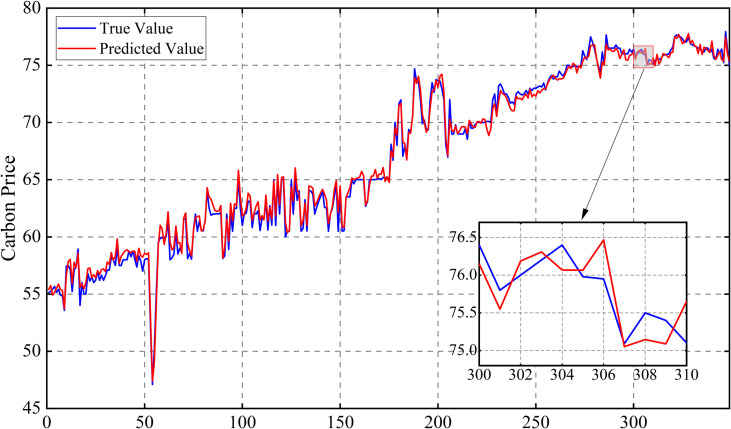
Results of carbon price forecasts for the Shanghai market.

Following this, the model is retrained using the optimized parameters and predictions are made on the test set. The prediction results of the three carbon markets are shown in Figs 13, 14 and 15, from which it can be observed that the prediction results are closer to the actual data, indicating the effectiveness of the model proposed in this paper.

**Fig 16:**
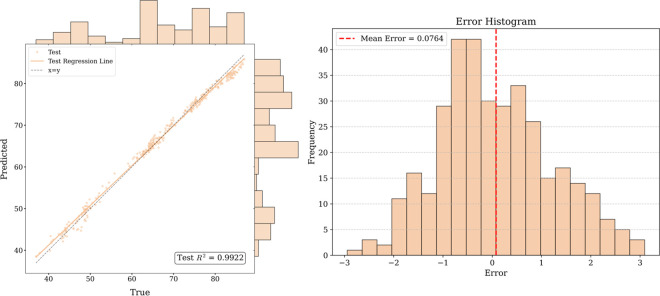
Fitted curve and error histogram of Guangdong carbon market.

**Fig 17:**
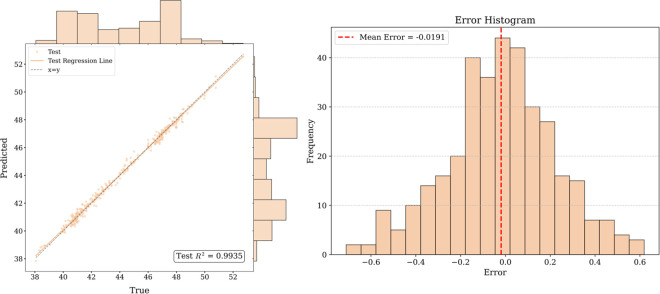
Fitted curve and error histogram of Hubei carbon market.

**Fig 18:**
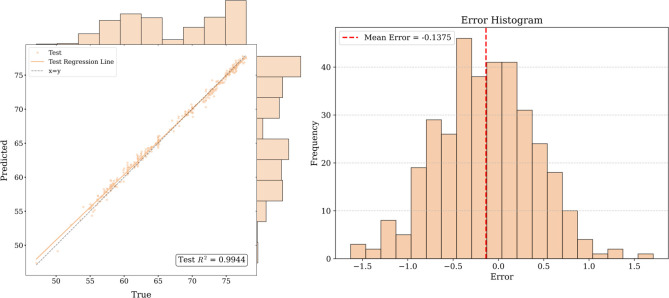
Fitted curve and error histogram of Shanghai carbon market.

Figs 16–17 and 18 show that for the prediction of low and high value regions, the model performs stably with an approximate normal distribution of errors and an average error close to 0, verifying the efficiency and reliability of the model.

### 4.6. Error evaluation and comparative analysis

**Fig 19:**
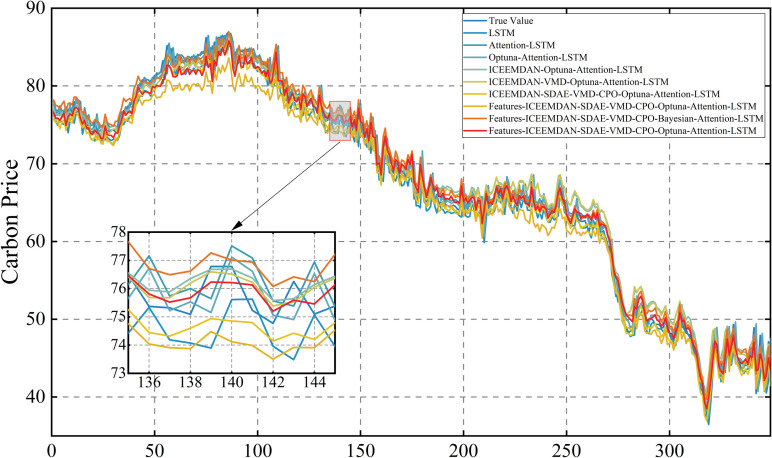
Comparison of multi-model forecasts of carbon price in Guangdong market.

**Fig 20:**
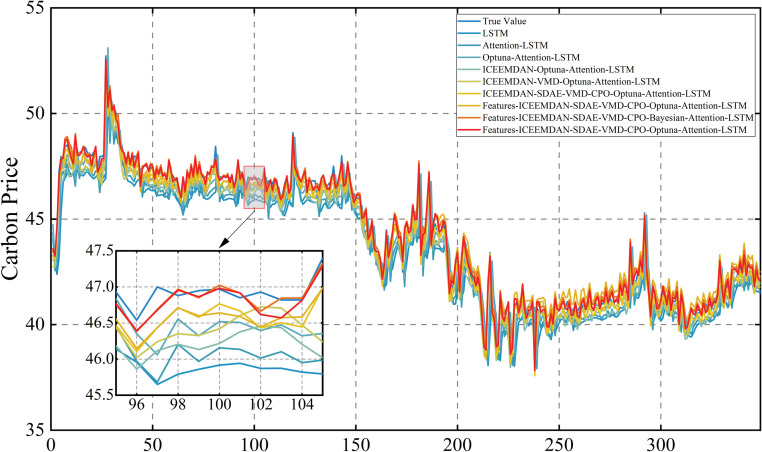
Comparison of multi-model forecasts of carbon price in Hubei market.

**Fig 21:**
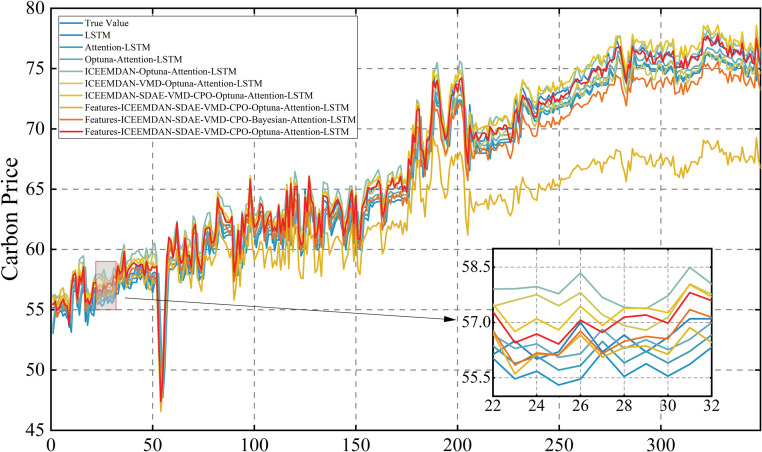
Comparison of multi-model forecasts of carbon price in Shanghai market.

In order to demonstrate the superiority of the proposed model, it was compared against eight benchmark models: LSTM, Attention-LSTM, Optuna-Attention-LSTM, ICEEMDAN-Optuna-Attention-LSTM, ICEEMDAN-VMD-Optuna-Attention-LSTM, ICEEMDAN-SDAE-VMD-CPO-Optuna-Attention-LSTM, Lasso-ICEEMDAN-SDAE-VMD-CPO-Optuna-Attention-LSTM, Features-ICEEMDAN-SDAE-VMD-CPO-Bayesian-Attention-LSTM. The model proposed in this study is Features-ICEEMDAN-SDAE-VMD-CPO-Optuna-Attention-LSTM. Attention-LSTM denotes the LSTM model that only incorporates the attention mechanism, and Optuna-Attention-LSTM performs Optuna parameter tuning of the LSTM on the basis of incorporating the attention mechanism, the ICEEMDAN-Optuna-Attention-LSTM adds a primary decomposition of the carbon price to the previous model, ICEEMDAN-VMD-Optuna-Attention-LSTM is a quadratic decomposition prediction hybrid model, ICEEMDAN-SDAE-VMD-CPO-Optuna-Attention-LSTM is the optimized quadratic decomposition prediction hybrid model, Lasso-ICEEMDAN-SDAE-VMD-CPO-Optuna-Attention-LSTM adds features selected by Lasso to the input of the previous model, while Features-ICEEMDAN-SDAE-VMD-CPO-Bayesian-Attention-LSTM adds features selected by comprehensive feature screening to the input, but uses Bayesian optimisation in the prediction process. As illustrated in Figs 19, 20 and 21, the prediction outcomes of the Features-ICEEMDAN-SDAE-VMD-CPO-Optuna-Attention-LSTM model closely align with actual values in most cases, indicating that the model has a strong prediction ability. In order to show the superiority of the model more intuitively, Tables 9–11 demonstrate the results of the prediction error assessment of various models on the test set. The results show that compared with the baseline model, the Features-ICEEMDAN-SDAE-VMD-CPO-Optuna-Attention-LSTM model has the smallest error values. In the three carbon markets, the four indicators of MSE, RMSE, MAE, and MAPE were reduced by 67.30%, 47.68%, 48.42%, and 48.79% on average, respectively. In addition, through gradual optimization, all stages of improvement measures significantly enhance the model’s prediction performance, and from the base model LSTM to the final model Features-ICEEMDAN-SDAE-VMD-CPO-Optuna-Attention-LSTM, the four error indicators showed a gradual downward trend in carbon price predictions for Guangdong, Hubei, and Shanghai, suggesting that the hybrid framework can significantly enhances model prediction accuracy through the synergistic effects of decomposition, noise reduction, parameter optimization, and fusion of external factors. It is further shown that the optimized quadratic decomposition prediction hybrid model with comprehensive feature screening proposed in this paper is reasonably effective in carbon price prediction, a finding consistent with the above conclusion.The parameter settings for the comparative experiments are detailed in Table 12.

**Table 9:** Comparison of the forecast results of the Guangdong carbon market.

Errors	MSE	RMSE	MAE	MAPE
LSTM	3.488	1.868	1.469	2.309
Attention-LSTM	2.974	1.724	1.270	2.108
Optuna-Attention-LSTM	2.842	1.686	1.220	2.016
ICEEMDAN-Optuna-Attention-LSTM	2.603	1.614	1.360	2.273
ICEEMDAN-VMD-Optuna-Attention-LSTM	2.160	1.470	1.233	2.040
ICEEMDAN-SDAE-VMD-CPO-Optuna-Attention-LSTM	1.527	1.236	1.025	1.529
Lasso-ICEEMDAN-SDAE-VMD-CPO-Optuna-Attention-LSTM	4.262	2.065	1.685	2.411
Features-ICEEMDAN-SDAE-VMD-CPO-Bayesian-Attention-LSTM	1.418	1.191	1.018	1.642
Features-ICEEMDAN-SDAE-VMD-CPO-Optuna-Attention-LSTM	1.327	1.152	0.933	1.436

**Table 10:** Comparison of the forecast results of the Hubei carbon market.

Errors	MSE	RMSE	MAE	MAPE
LSTM	1.477	1.215	0.998	2.239
Attention-LSTM	1.295	1.138	0.908	2.039
Optuna-Attention-LSTM	0.917	0.957	0.669	1.507
ICEEMDAN-Optuna-Attention-LSTM	0.504	0.710	0.623	1.393
ICEEMDAN-VMD-Optuna-Attention-LSTM	0.269	0.519	0.423	0.939
ICEEMDAN-SDAE-VMD-CPO-Optuna-Attention-LSTM	0.158	0.398	0.343	0.772
Lasso-ICEEMDAN-SDAE-VMD-CPO-Optuna-Attention-LSTM	0.187	0.432	0.359	0.827
Features-ICEEMDAN-SDAE-VMD-CPO-Bayesian-Attention-LSTM	0.084	0.290	0.228	0.524
Features-ICEEMDAN-SDAE-VMD-CPO-Optuna-Attention-LSTM	0.060	0.245	0.192	0.436

**Table 11:** Comparison of the forecast results of the Shanghai carbon market.

Errors	MSE	RMSE	MAE	MAPE
LSTM	3.375	1.837	1.495	2.267
Attention-LSTM	2.822	1.680	1.308	1.985
Optuna-Attention-LSTM	2.297	1.516	1.074	1.653
ICEEMDAN-Optuna-Attention-LSTM	1.315	1.147	0.952	1.501
ICEEMDAN-VMD-Optuna-Attention-LSTM	1.094	1.046	0.881	1.312
ICEEMDAN-SDAE-VMD-CPO-Optuna-Attention-LSTM	0.710	0.842	0.764	1.157
Lasso-ICEEMDAN-SDAE-VMD-CPO-Optuna-Attention-LSTM	30.112	5.487	4.527	6.341
Features-ICEEMDAN-SDAE-VMD-CPO-Bayesian-Attention-LSTM	2.244	1.498	1.206	1.692
Features-ICEEMDAN-SDAE-VMD-CPO-Optuna-Attention-LSTM	0.304	0.551	0.440	0.680

**Table 12:** Parameter settings for the comparative experiment.

Reference model	Parameter category	Parameter values
LSTM	LSTM units	50
	dropout rate	0.2
Attention-LSTM	LSTM units	50
	dropout rate	0.2
Optuna-Attention-LSTM	LSTM units	[10,100]
	dropout rate	[0.1,0.5]
ICEEMDAN-Optuna-Attention-LSTM	LSTM units	[10,100]
	dropout rate	[0.1,0.5]
ICEEMDAN-VMD-Optuna-Attention-LSTM	LSTM units	[10,100]
	dropout rate	[0.1,0.5]
ICEEMDAN-SDAE-VMD-CPO-Optuna-Attention-LSTM	LSTM units	[10,100]
	dropout rate	[0.1,0.5]
Lasso-ICEEMDAN-SDAE-VMD-CPO-Optuna-Attention-LSTM	LSTM units	[10,100]
	dropout rate	[0.1,0.5]
Features-ICEEMDAN-SDAE-VMD-CPO-Bayesian-Attention-LSTM	LSTM units	[10,100]
	dropout rate	[0.1,0.5]
Features-ICEEMDAN-SDAE-VMD-CPO-Optuna-Attention-LSTM	LSTM units	[10,100]
	dropout rate	[0.1,0.5]

In this comparative experiment, although the basic LSTM model can capture the temporal dependencies in time series, it lacks the ability to identify and focus on key information, resulting in limited prediction accuracy. After introducing the attention mechanism, the model can focus on important time step information in a targeted manner, better adapting to the uneven importance distribution characteristic of carbon emission allowance price fluctuations. Through hyperparameter optimisation using the optuna framework, the LSTM units and dropout rate can be further optimised to effectively suppress overfitting. On this basis, ICEEMDAN decomposition was used to decompose the carbon emission quota price sequence, effectively capturing different frequency components. Further noise reduction of the high-frequency components using SDAE better preserved key information. Subsequently, VMD was introduced to refine the signal decomposition and improve the accuracy of feature extraction. Next, features were selected using a comprehensive feature screening method that took into account both non-linear and linear features, thereby retaining key variables while effectively reducing the data dimension. Finally, the Features-ICEEMDAN-SDAE-VMD-CPO-Optuna-Attention-LSTM model demonstrates the best prediction performance.

### 4.7. Model interpretability analysis

We use SHAP analysis to explain the decision-making process in the model. SHAP Comprehensive charts clearly show the contribution of selected features to the model’s prediction results.

**Fig 22:**
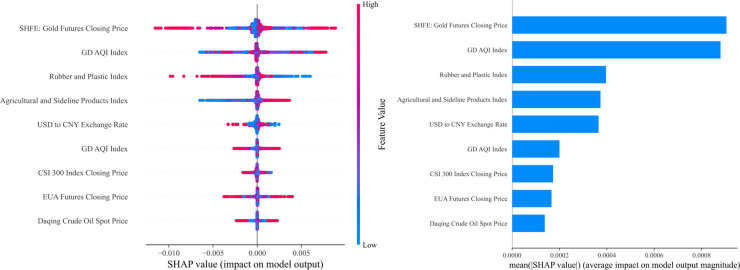
Comprehensive analysis chart of SHAP values in the Guangdong carbon market.

**Fig 23:**
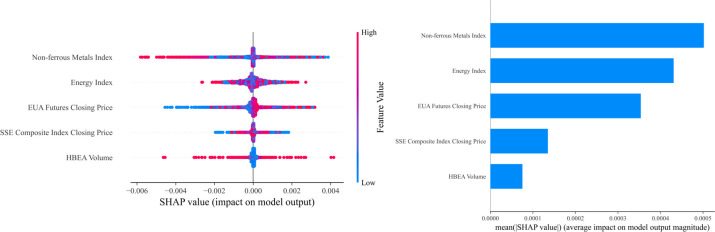
Comprehensive analysis chart of SHAP values in the Hubei carbon market.

**Fig 24:**
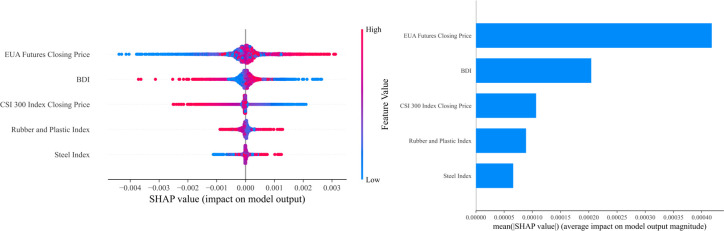
Comprehensive analysis chart of SHAP values in the Shanghai carbon market.

As shown in Figs 22, 23 and 24, In the Guangdong carbon market, the SHFE: Gold futures closing price and GD AQI index contribute the most to the prediction results, indicating that macroeconomic conditions and regional environmental status play a core role in carbon market pricing. Additionally, the Rubber and plastic index and Agricultural and sideline products index also hold significant importance. Furthermore, changes in the USD to CNY exchange rate and CSI 300 index closing price also influence the trend of carbon emission rights prices to some extent. In contrast, EUA futures closing price and Daqing crude oil spot price have a relatively minor impact on the Guangdong carbon market.

In the Hubei carbon market, the Energy index is the most critical influencing factor, contributing the most to fluctuations in carbon emission rights prices, reflecting the direct driving role of energy market changes on carbon market prices. Secondly, the EUA futures closing price and the Non-ferrous metals index also demonstrate significant influence, indicating that the performance of the international carbon trading market and industrial production activities have a significant linkage effect on local carbon emission rights prices. Additionally, the SSE composite index closing price also influences carbon emission rights prices to a certain extent. In contrast, the HBEA volume has a relatively minor direct impact on carbon emission rights prices.

In the Shanghai carbon market, the EUA futures closing price is the most important influencing factor, indicating that global carbon market changes have a direct and significant driving effect on Shanghai carbon market prices. The BDI also has high importance, reflecting the indirect link between international trade activity levels and carbon emission demand. The CSI 300 index closing price also exhibits strong influence, indicating that the macroeconomic environment plays a key role in the carbon emission rights market. Additionally, the Rubber and plastic index and Steel index also exert a certain influence on the forecast results. Overall, Shanghai carbon emission rights prices are jointly influenced by global carbon market dynamics and local economic conditions.

## 5. Conclusion

Accurately predicting carbon prices not only aids market participants make better decisions, but also promotes the stable evolution of the carbon market. Therefore, this study proposes an optimized quadratic decomposition forecasting hybrid model with comprehensive feature screening, combining the advantages of ICEEMDAN, SDAE, VMD, CPO, Optuna, Attention, and LSTM. The model integrates internal factors and external influences on carbon price in the final input, and innovatively incorporates domestic industry factors into the structural factors. Empirical validation in three carbon markets, Guangdong, Hubei and Shanghai, and comparison with the benchmark model show that the proposed model has superior forecasting performance. The findings of this paper are as follows: (1) The comprehensive feature screening approach not only combines the advantages of nonlinear and linear feature selection, but also improves the robustness, generalization ability and interpretability of carbon price forecasting. (2) Introducing external influences into the decomposition and reconstruction framework can capture the direct effects of external changes on carbon prices more accurately, thus improving the prediction accuracy of the model. (3) The optimized secondary decomposition technique can decompose the original signal of carbon price more thoroughly and remove the residual noise, which provides a stable and reliable foundation for the subsequent prediction model. (4) The Optuna-Attention-LSTM model proposed in this paper performs well, avoids problems such as insufficient weighting of key features, effectively solves the inefficiency of traditional tuning and meets the demand for automated tuning.

Although the predictive hybrid model proposed in this study performs well, it still has certain limitations. First, with the continuous development of subsequent models and algorithms, the predictive performance can be further improved by introducing more advanced methods. Second, the applicability of this model needs to be further expanded. This study only predicts the next time step of carbon prices and does not consider long-term trends. Additionally, the model framework does not account for the impact of policy changes or extreme weather events. Future research could incorporate economic policy uncertainty and climate policy uncertainty to explore their effects on model predictions. However, since policy uncertainty data is primarily monthly, it remains to be explored whether a mixed-frequency model is suitable for predicting daily carbon prices.

## Supporting information

S1 Data Code and code description. (ZIP)
