# Supplementary figures and images for: Forecasting regional carbon prices in china with a hybrid model based on quadratic decomposition and comprehensive feature screening
Source: PLoS One. 2025 Jun 30;20(6):e0326926. doi: 10.1371/journal.pone.0326926 (PMC12208498; doi:10.1371/journal.pone.0326926)

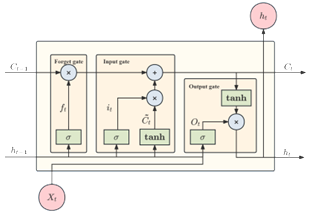

Supplement: S1 Data — (ZIP) [file pone.0326926.s001.zip › supplementary information/supplementary information/High-Definition Original Images in the Manuscript/Fig 1.tif]

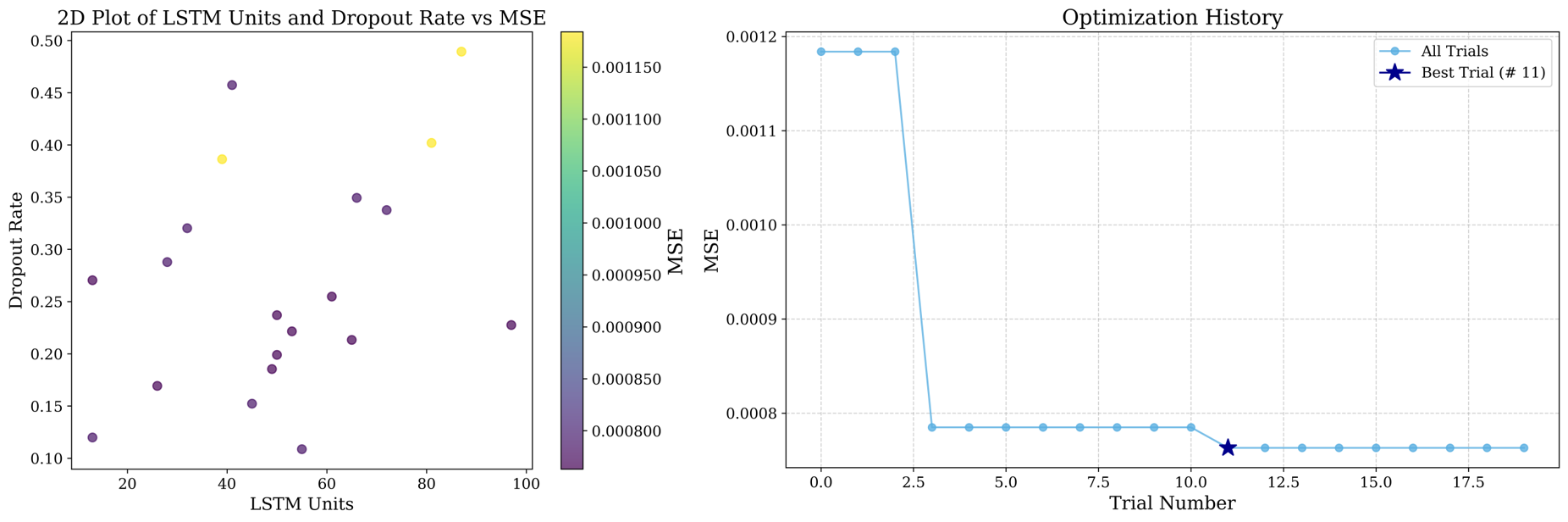

Supplement: S1 Data — (ZIP) [file pone.0326926.s001.zip › supplementary information/supplementary information/High-Definition Original Images in the Manuscript/Fig 10.tif]

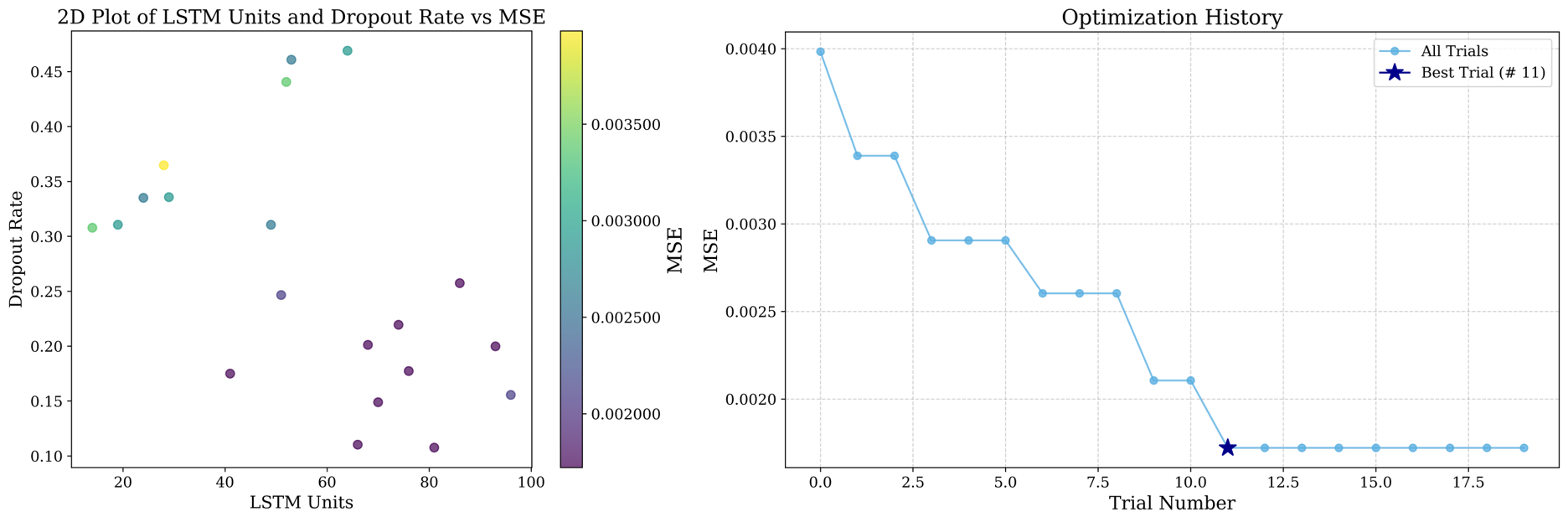

Supplement: S1 Data — (ZIP) [file pone.0326926.s001.zip › supplementary information/supplementary information/High-Definition Original Images in the Manuscript/Fig 11.tif]

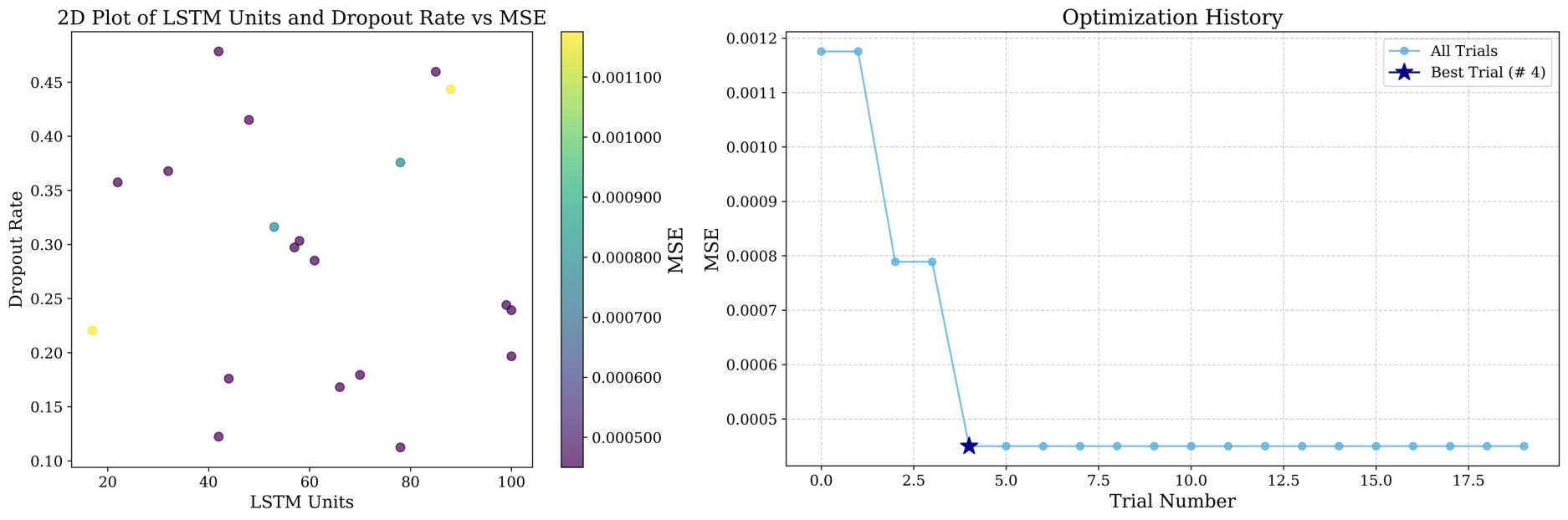

Supplement: S1 Data — (ZIP) [file pone.0326926.s001.zip › supplementary information/supplementary information/High-Definition Original Images in the Manuscript/Fig 12.tif]

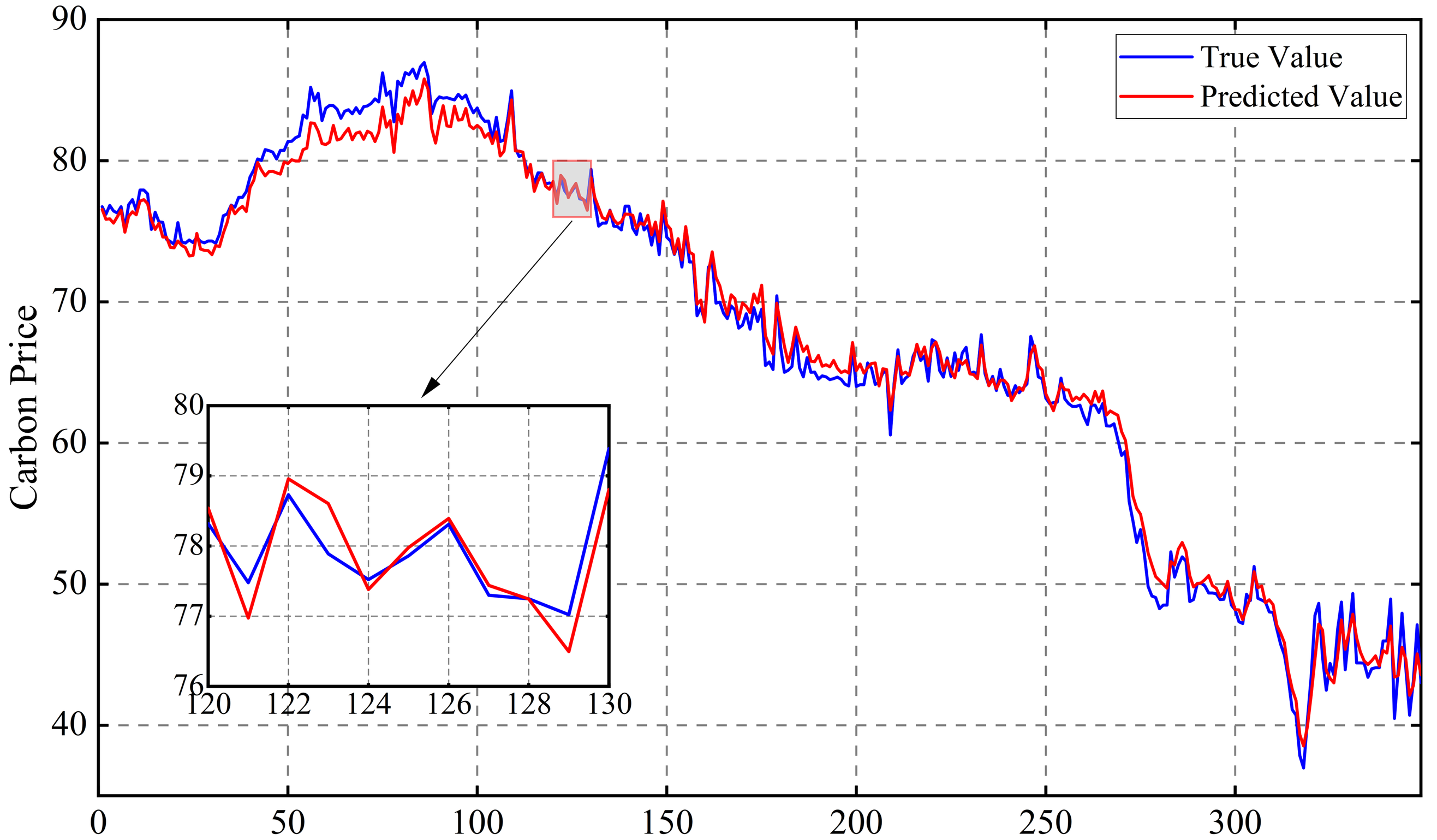

Supplement: S1 Data — (ZIP) [file pone.0326926.s001.zip › supplementary information/supplementary information/High-Definition Original Images in the Manuscript/Fig 13.tif]

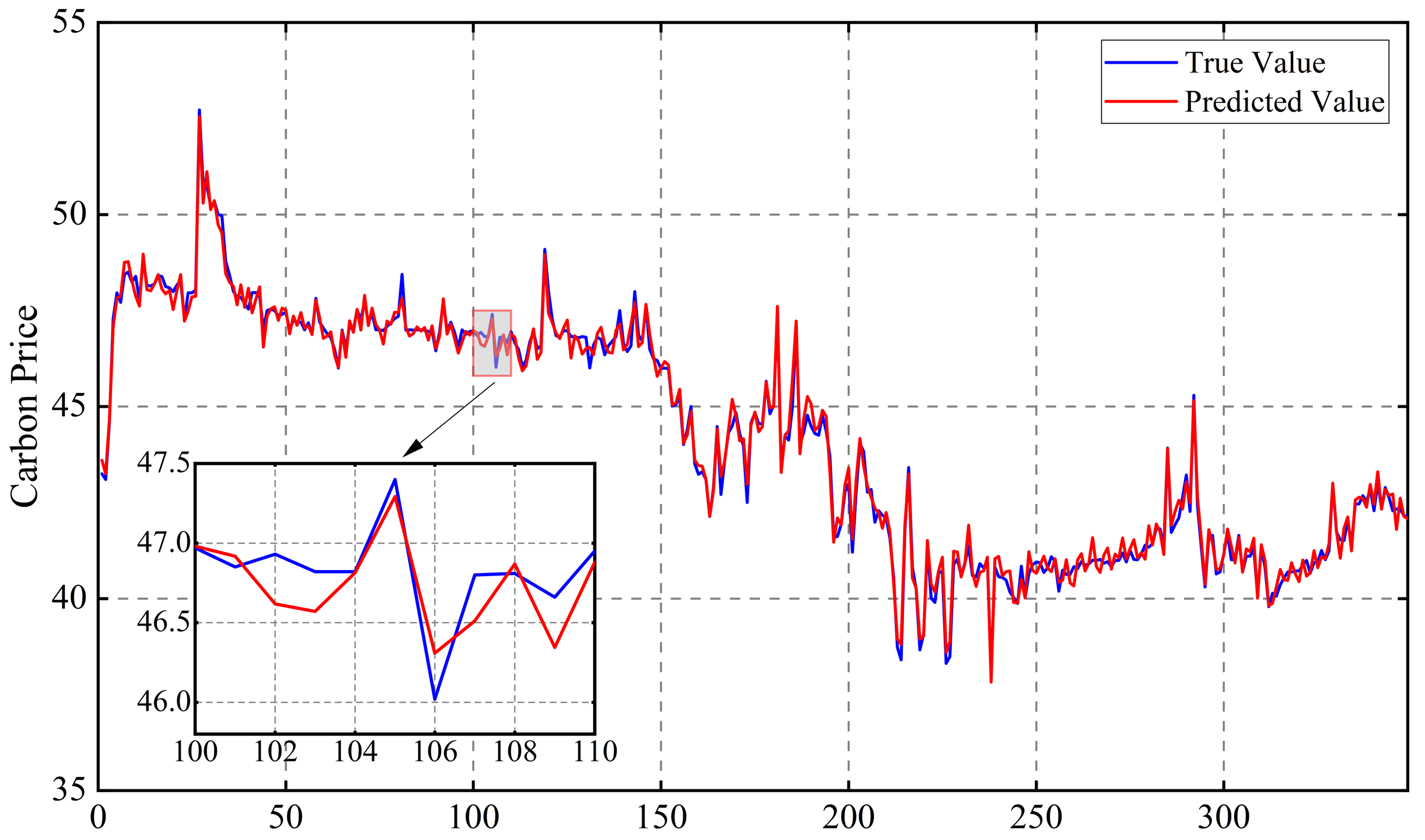

Supplement: S1 Data — (ZIP) [file pone.0326926.s001.zip › supplementary information/supplementary information/High-Definition Original Images in the Manuscript/Fig 14.tif]

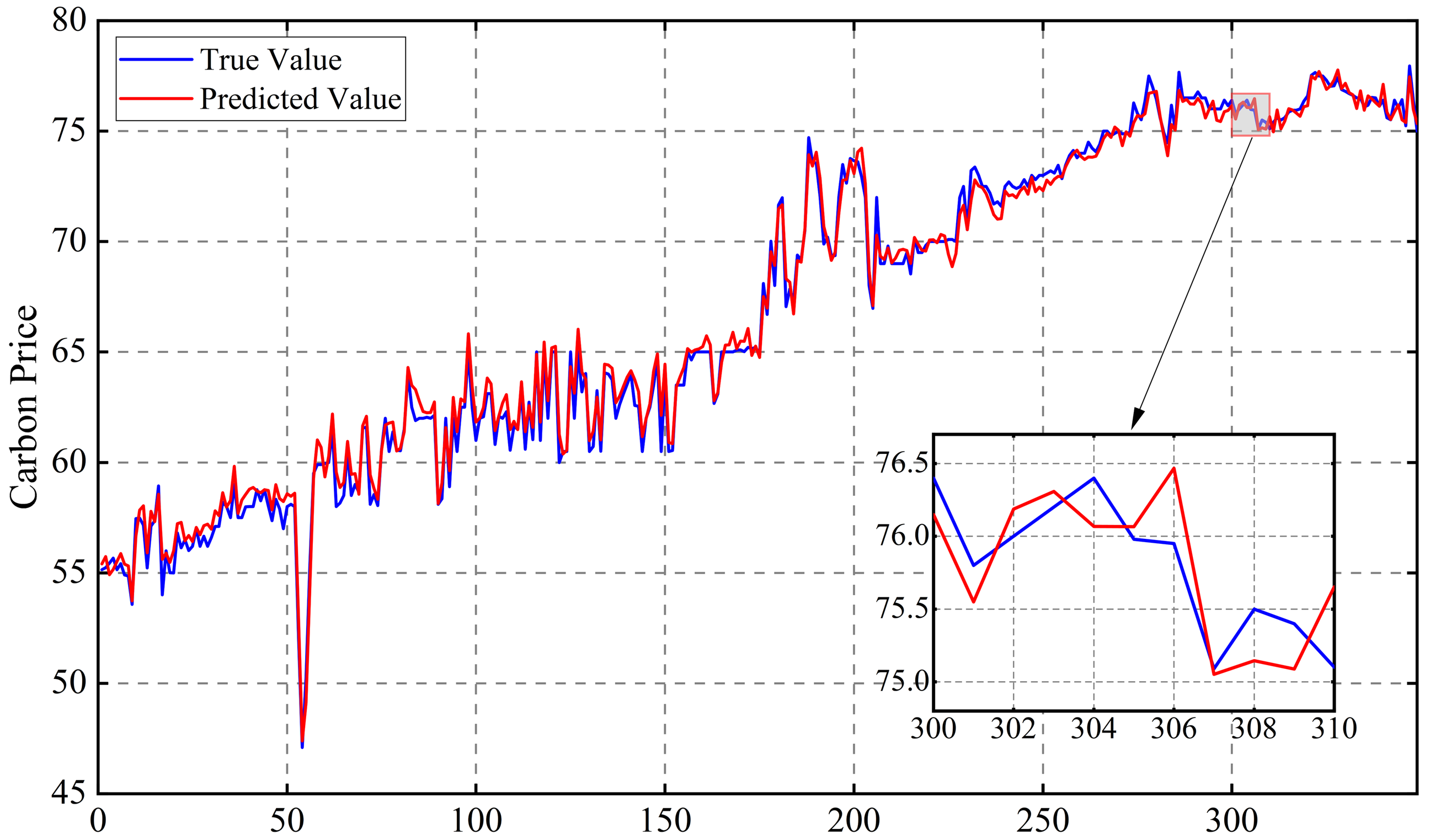

Supplement: S1 Data — (ZIP) [file pone.0326926.s001.zip › supplementary information/supplementary information/High-Definition Original Images in the Manuscript/Fig 15.tif]

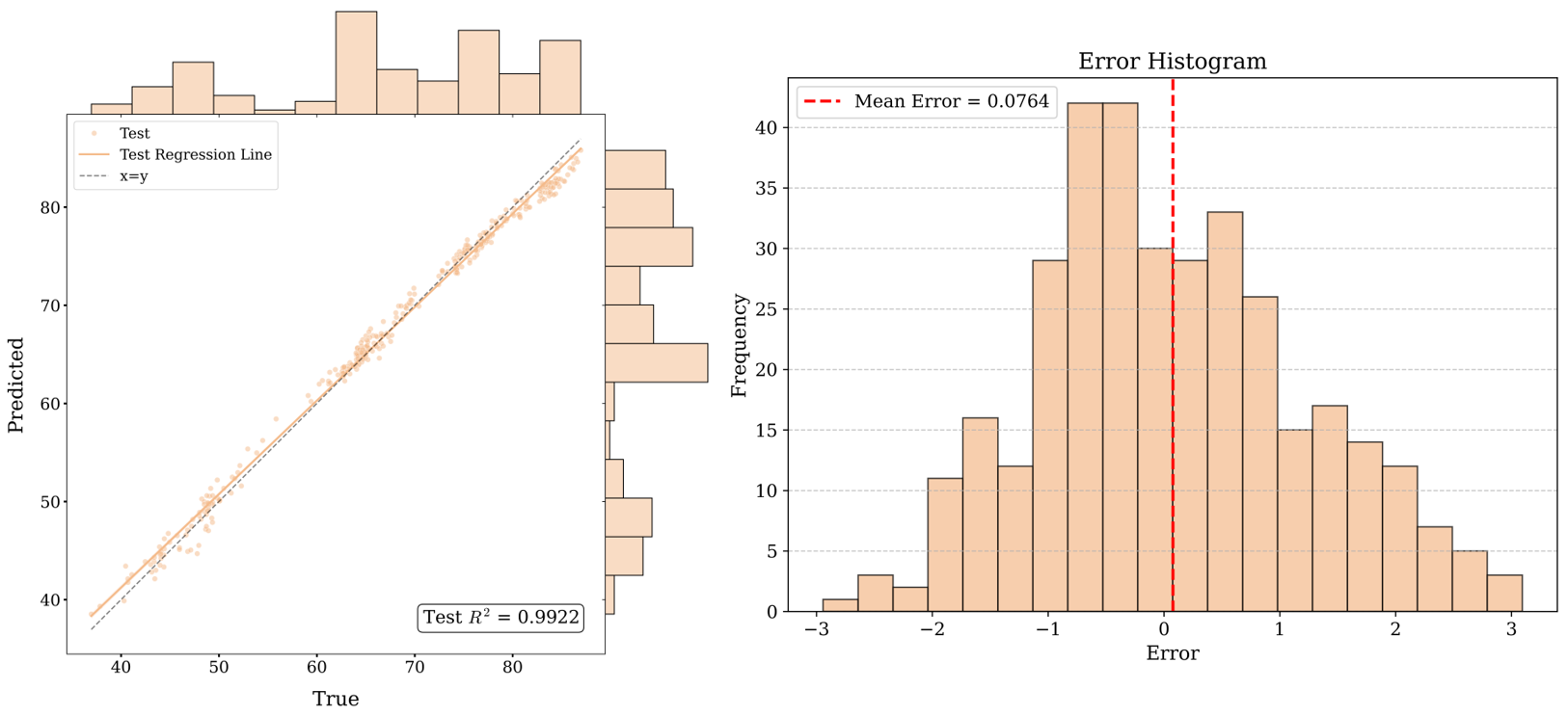

Supplement: S1 Data — (ZIP) [file pone.0326926.s001.zip › supplementary information/supplementary information/High-Definition Original Images in the Manuscript/Fig 16.tif]

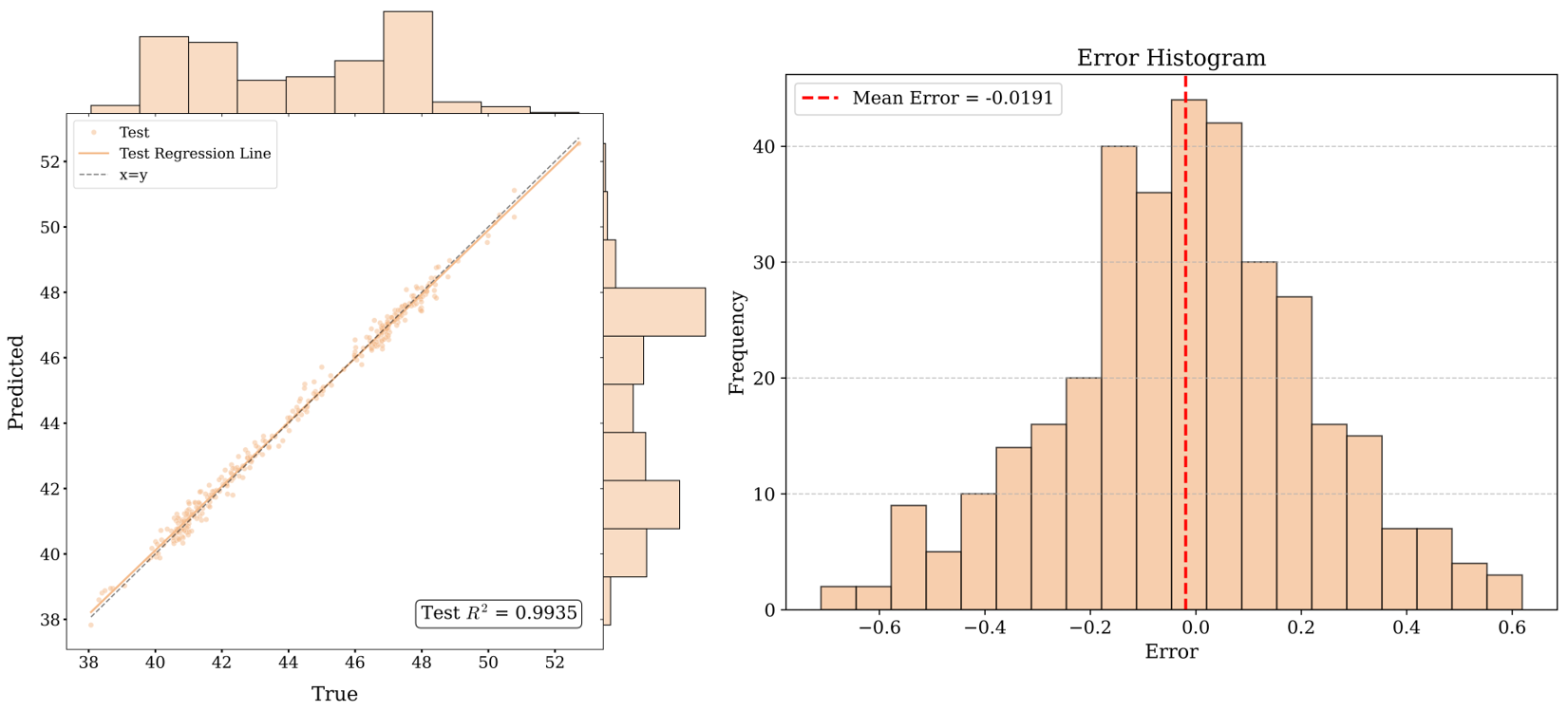

Supplement: S1 Data — (ZIP) [file pone.0326926.s001.zip › supplementary information/supplementary information/High-Definition Original Images in the Manuscript/Fig 17.tif]

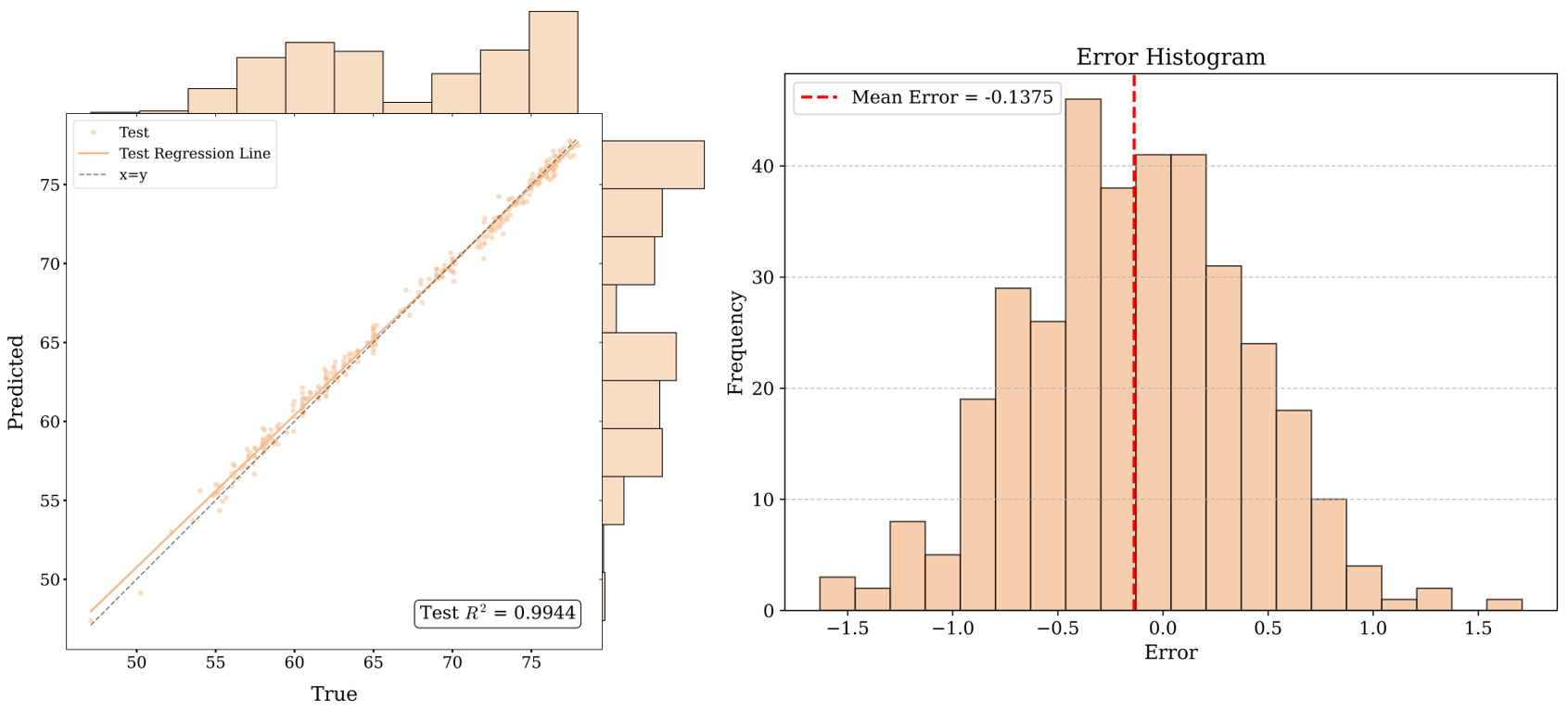

Supplement: S1 Data — (ZIP) [file pone.0326926.s001.zip › supplementary information/supplementary information/High-Definition Original Images in the Manuscript/Fig 18.tif]

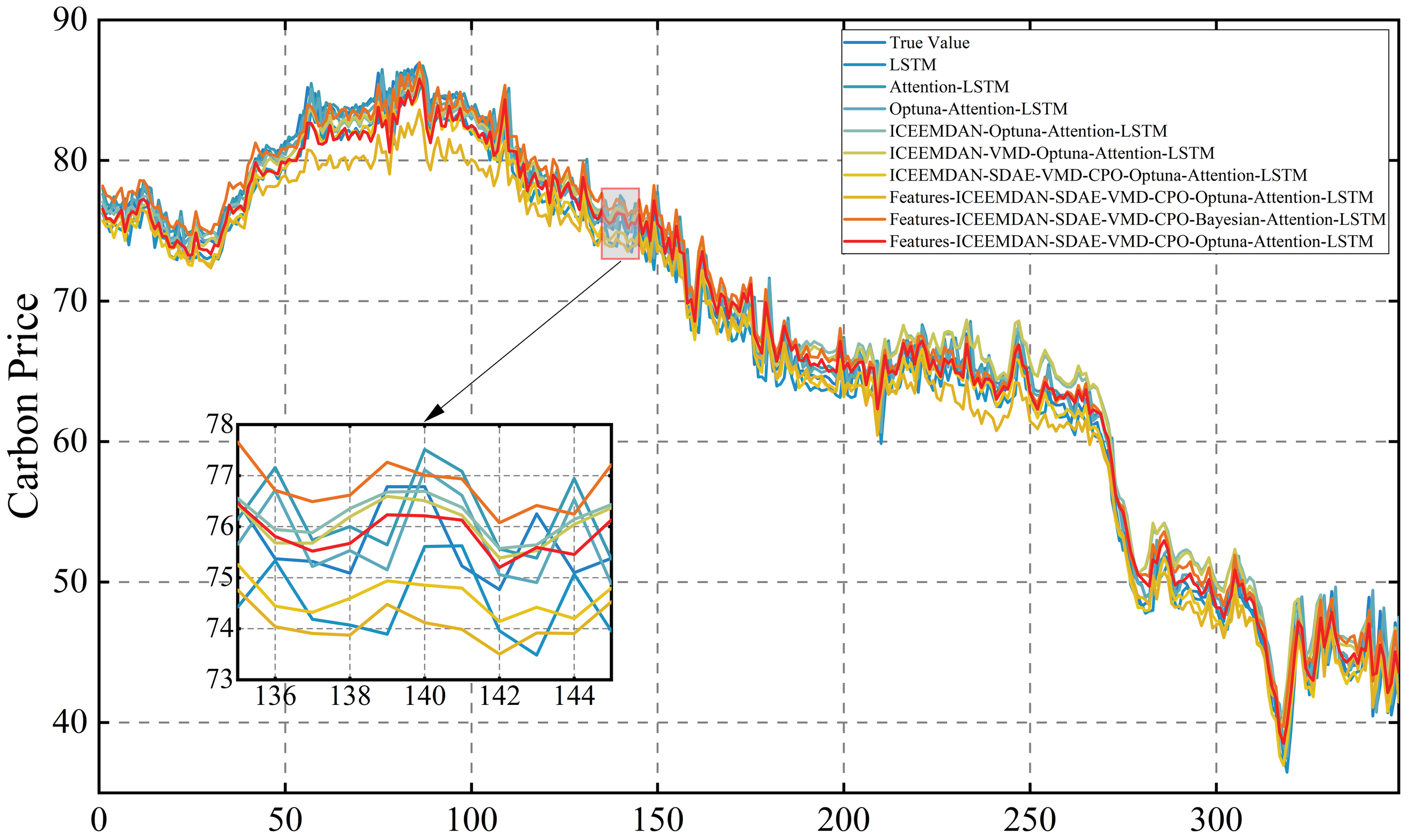

Supplement: S1 Data — (ZIP) [file pone.0326926.s001.zip › supplementary information/supplementary information/High-Definition Original Images in the Manuscript/Fig 19.tif]

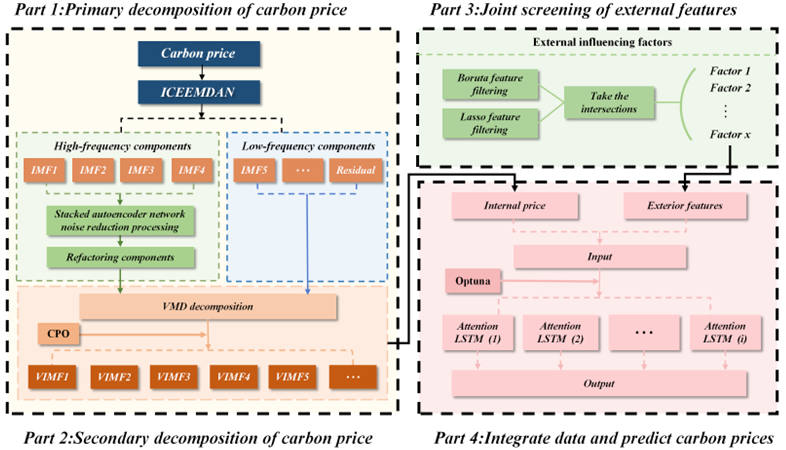

Supplement: S1 Data — (ZIP) [file pone.0326926.s001.zip › supplementary information/supplementary information/High-Definition Original Images in the Manuscript/Fig 2.tif]

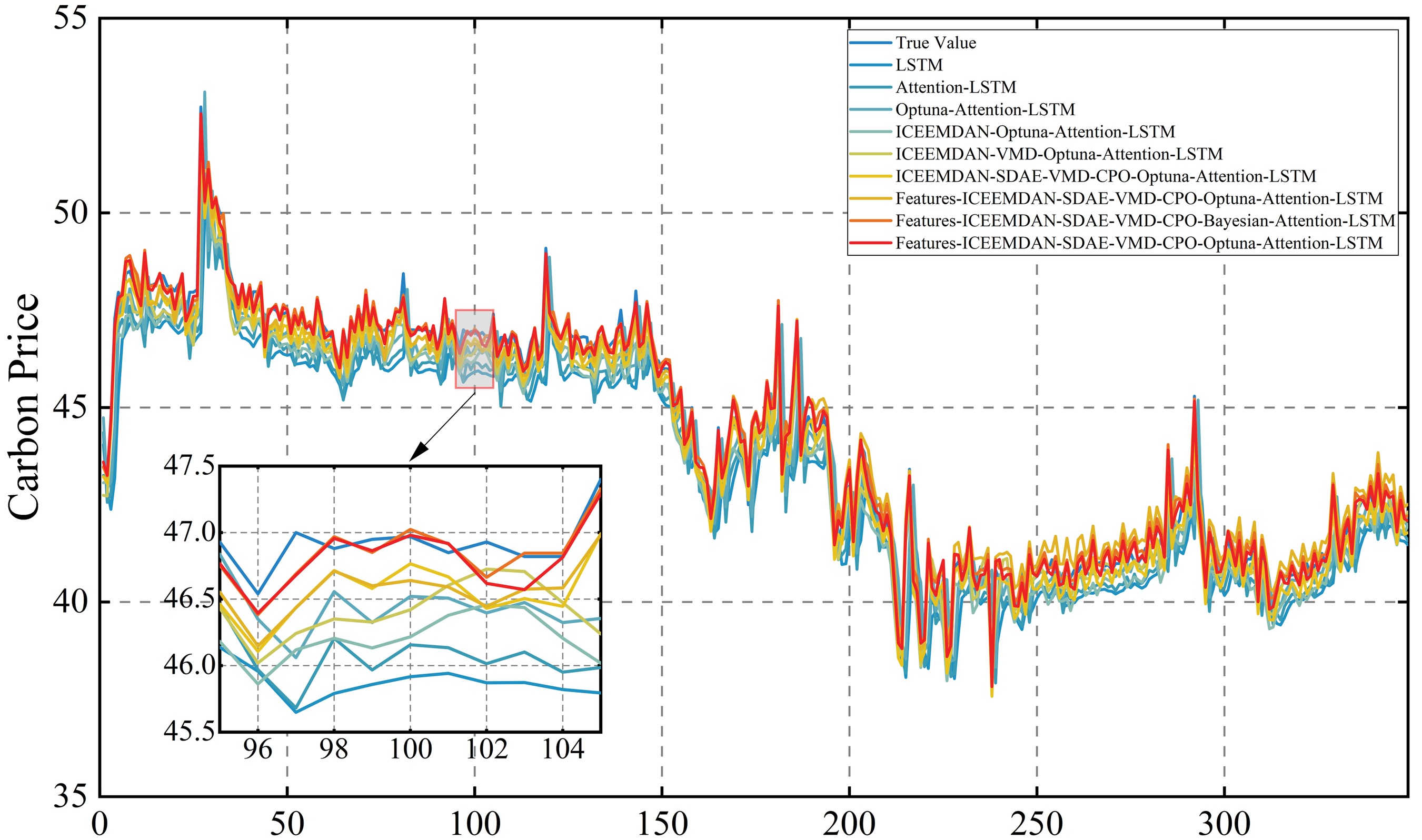

Supplement: S1 Data — (ZIP) [file pone.0326926.s001.zip › supplementary information/supplementary information/High-Definition Original Images in the Manuscript/Fig 20.tif]

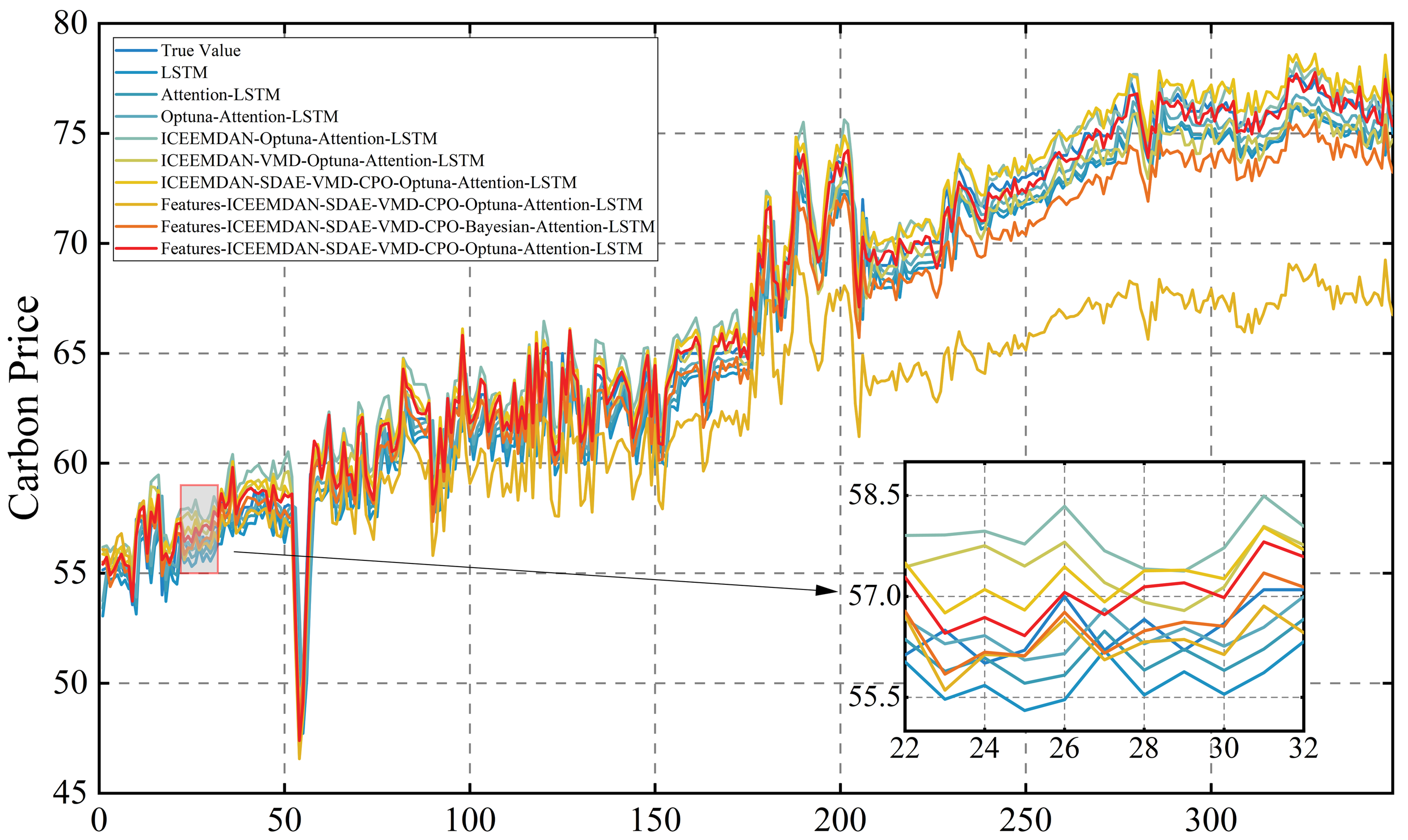

Supplement: S1 Data — (ZIP) [file pone.0326926.s001.zip › supplementary information/supplementary information/High-Definition Original Images in the Manuscript/Fig 21.tif]

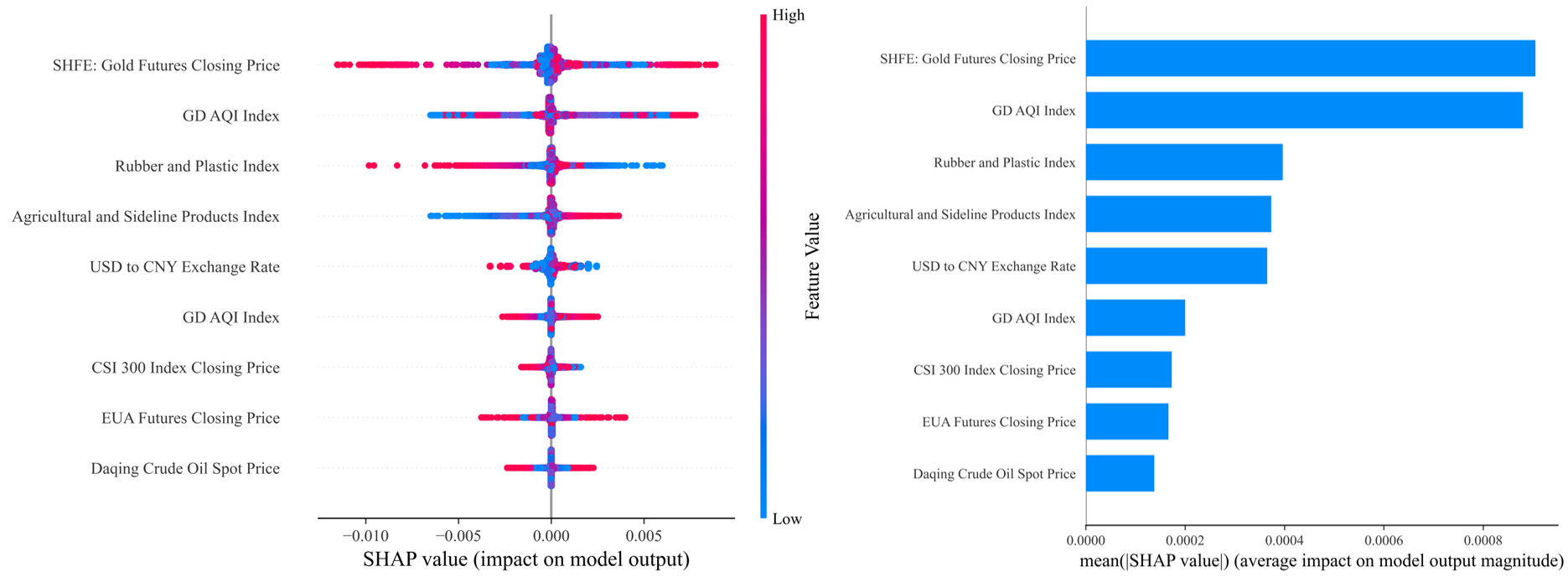

Supplement: S1 Data — (ZIP) [file pone.0326926.s001.zip › supplementary information/supplementary information/High-Definition Original Images in the Manuscript/Fig 22.tif]

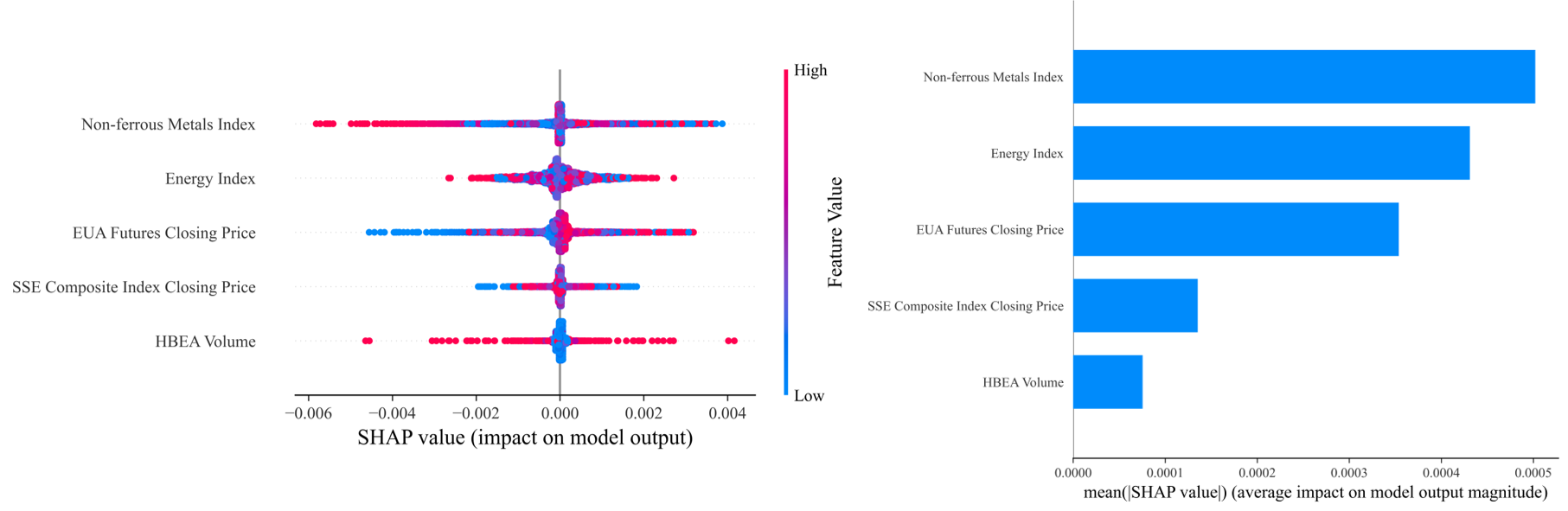

Supplement: S1 Data — (ZIP) [file pone.0326926.s001.zip › supplementary information/supplementary information/High-Definition Original Images in the Manuscript/Fig 23.tif]

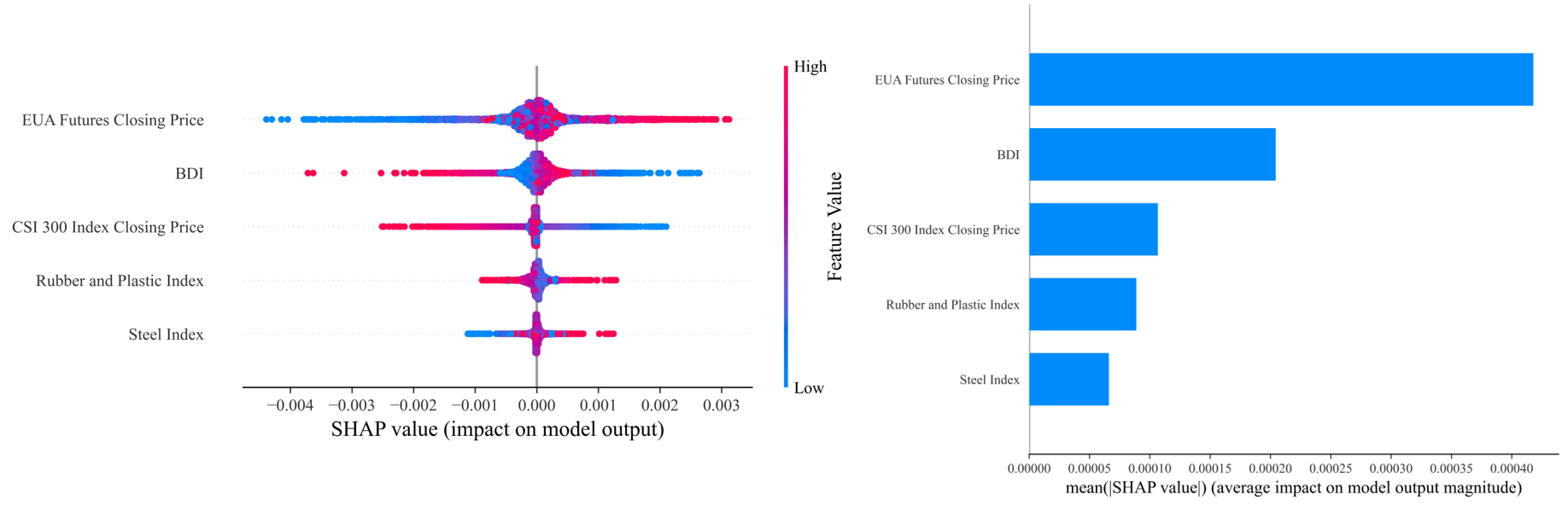

Supplement: S1 Data — (ZIP) [file pone.0326926.s001.zip › supplementary information/supplementary information/High-Definition Original Images in the Manuscript/Fig 24.tif]

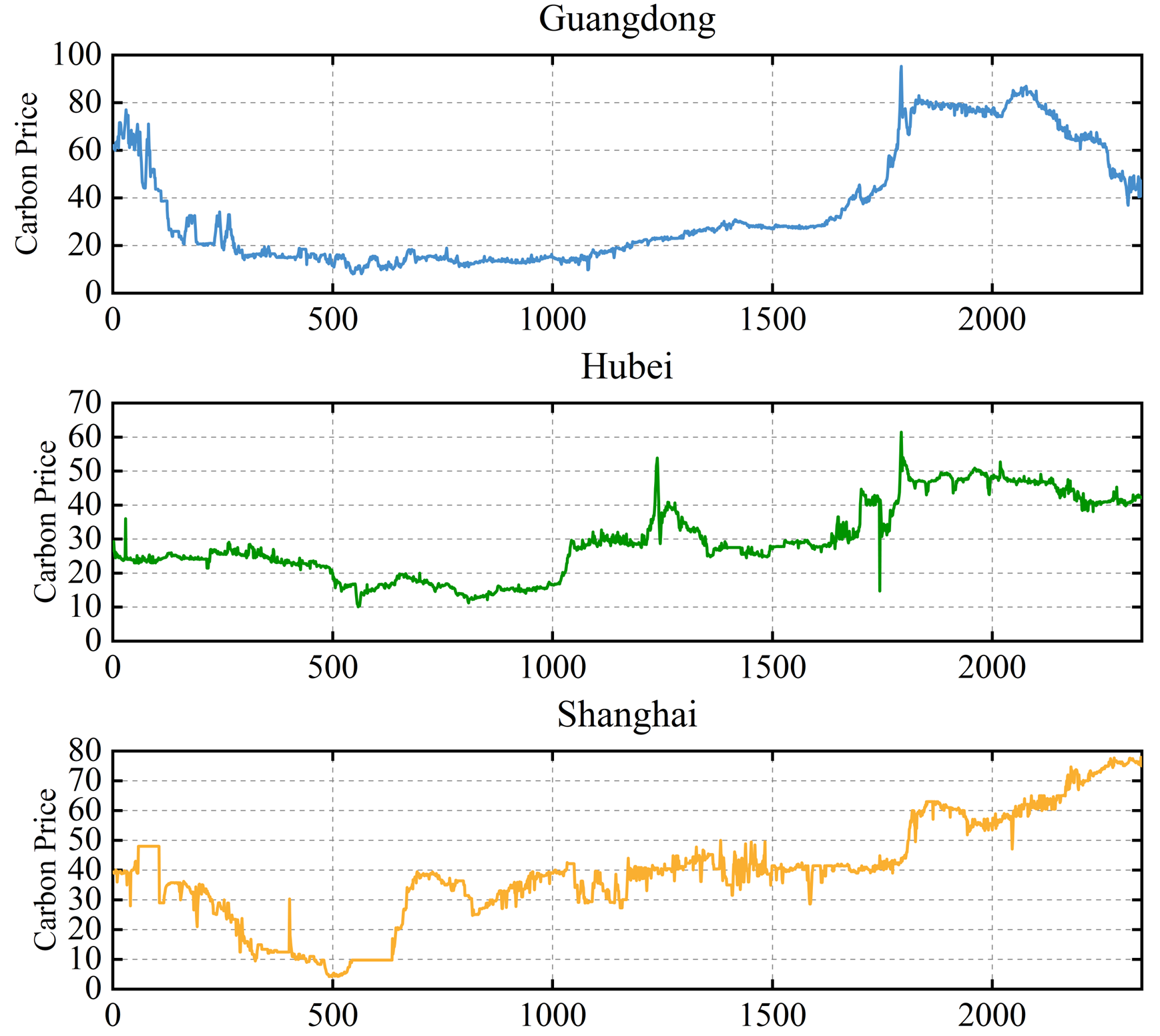

Supplement: S1 Data — (ZIP) [file pone.0326926.s001.zip › supplementary information/supplementary information/High-Definition Original Images in the Manuscript/Fig 3.tif]

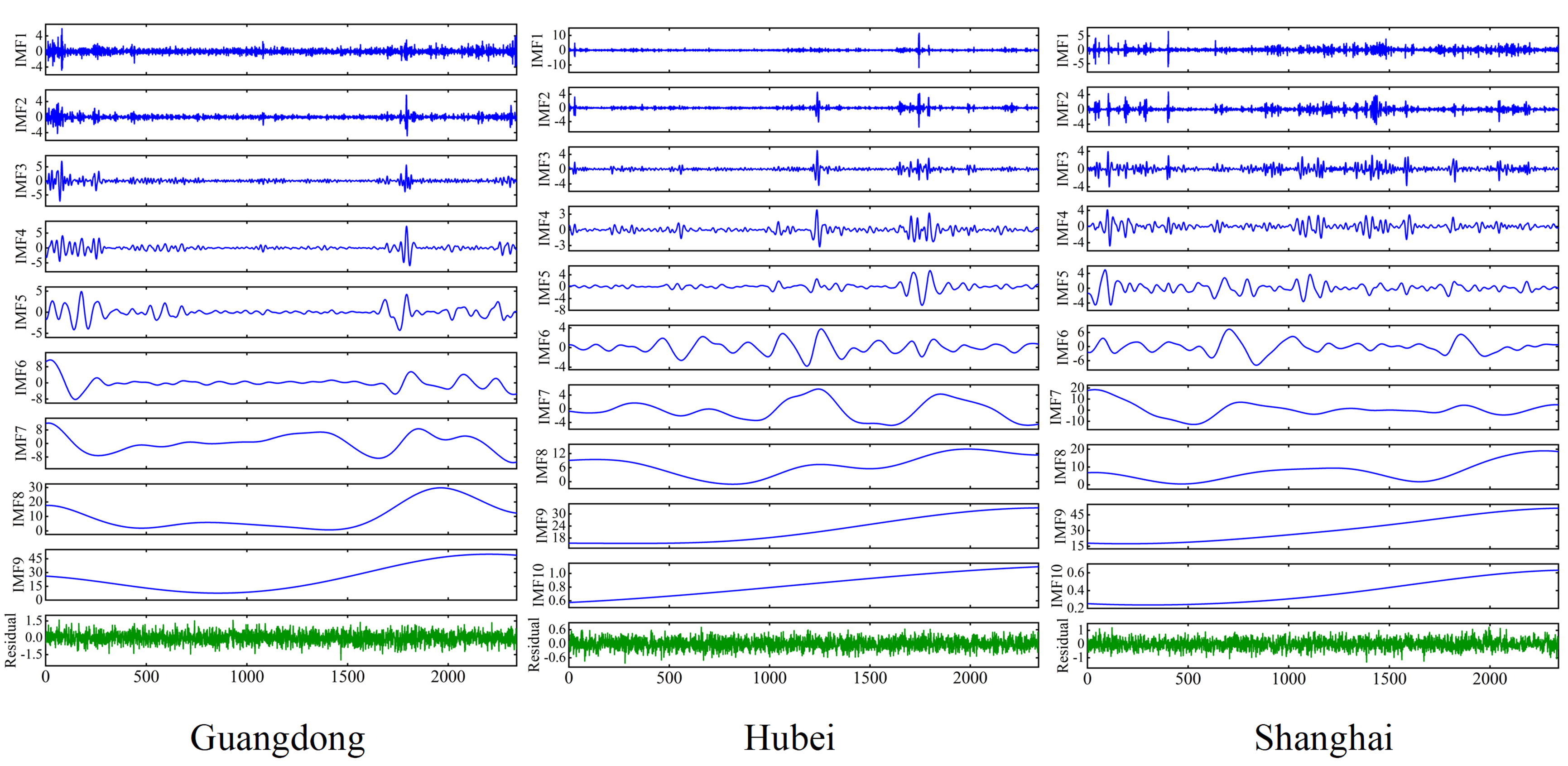

Supplement: S1 Data — (ZIP) [file pone.0326926.s001.zip › supplementary information/supplementary information/High-Definition Original Images in the Manuscript/Fig 4.tif]

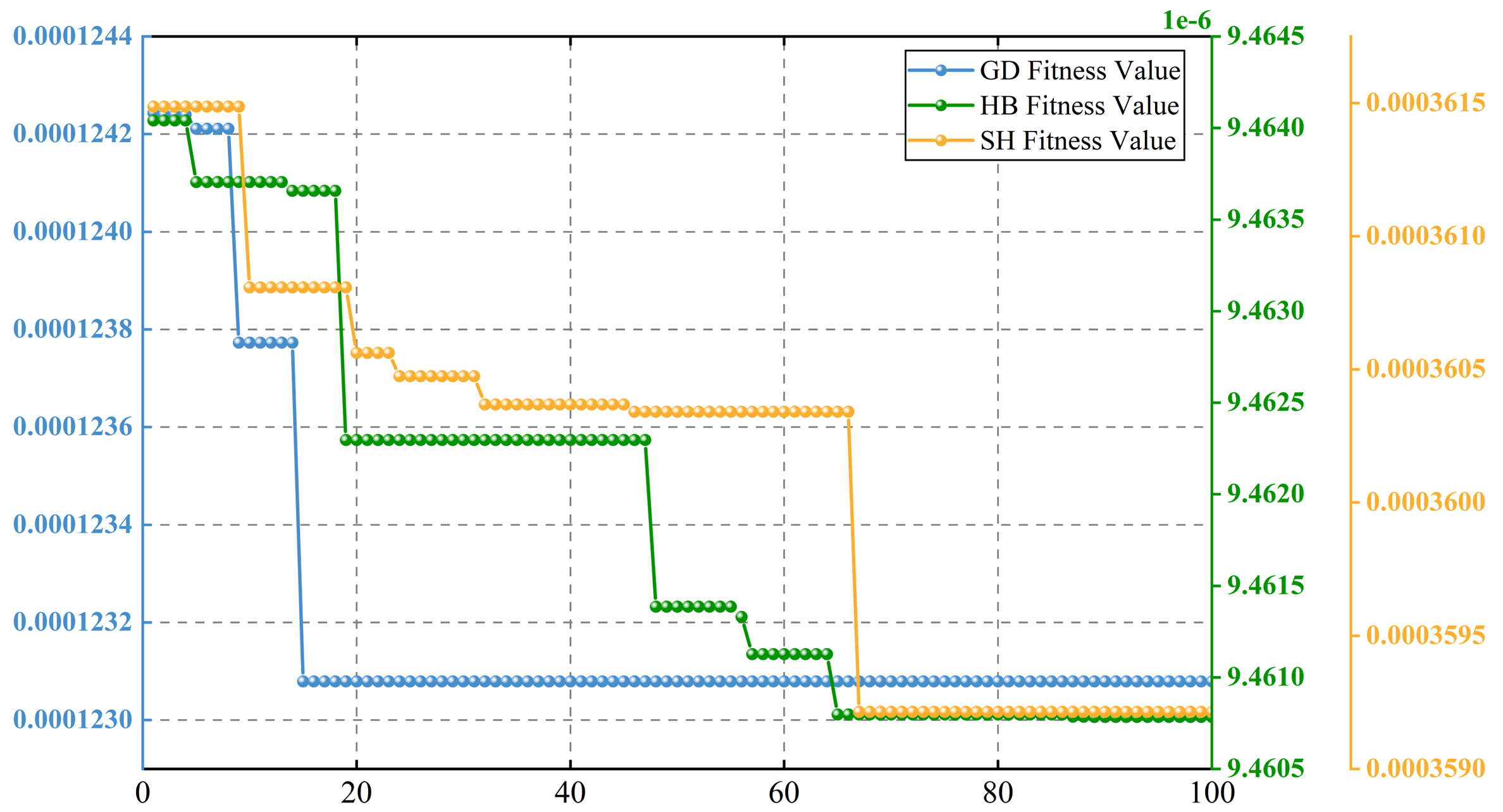

Supplement: S1 Data — (ZIP) [file pone.0326926.s001.zip › supplementary information/supplementary information/High-Definition Original Images in the Manuscript/Fig 5.tif]

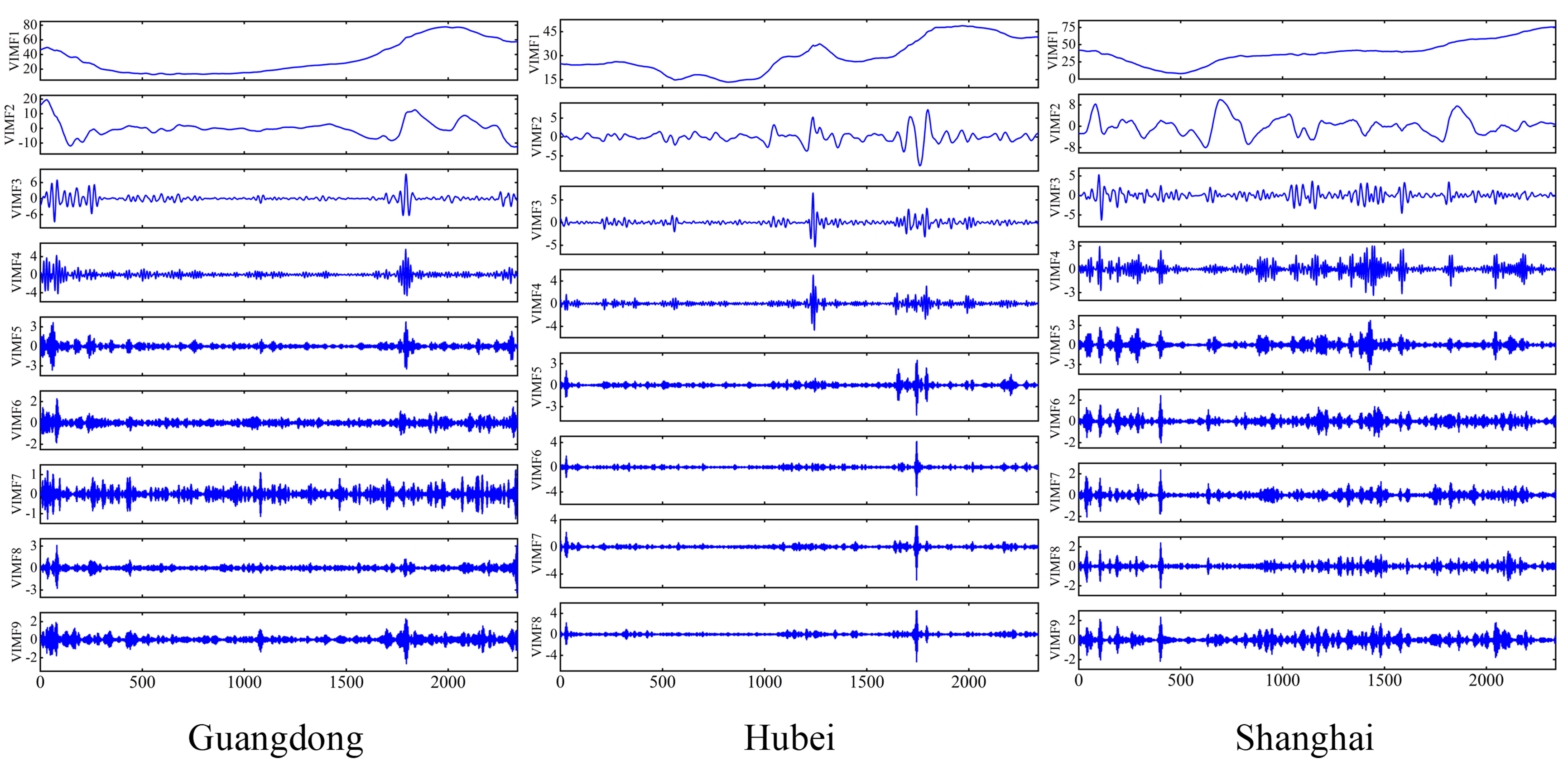

Supplement: S1 Data — (ZIP) [file pone.0326926.s001.zip › supplementary information/supplementary information/High-Definition Original Images in the Manuscript/Fig 6.tif]

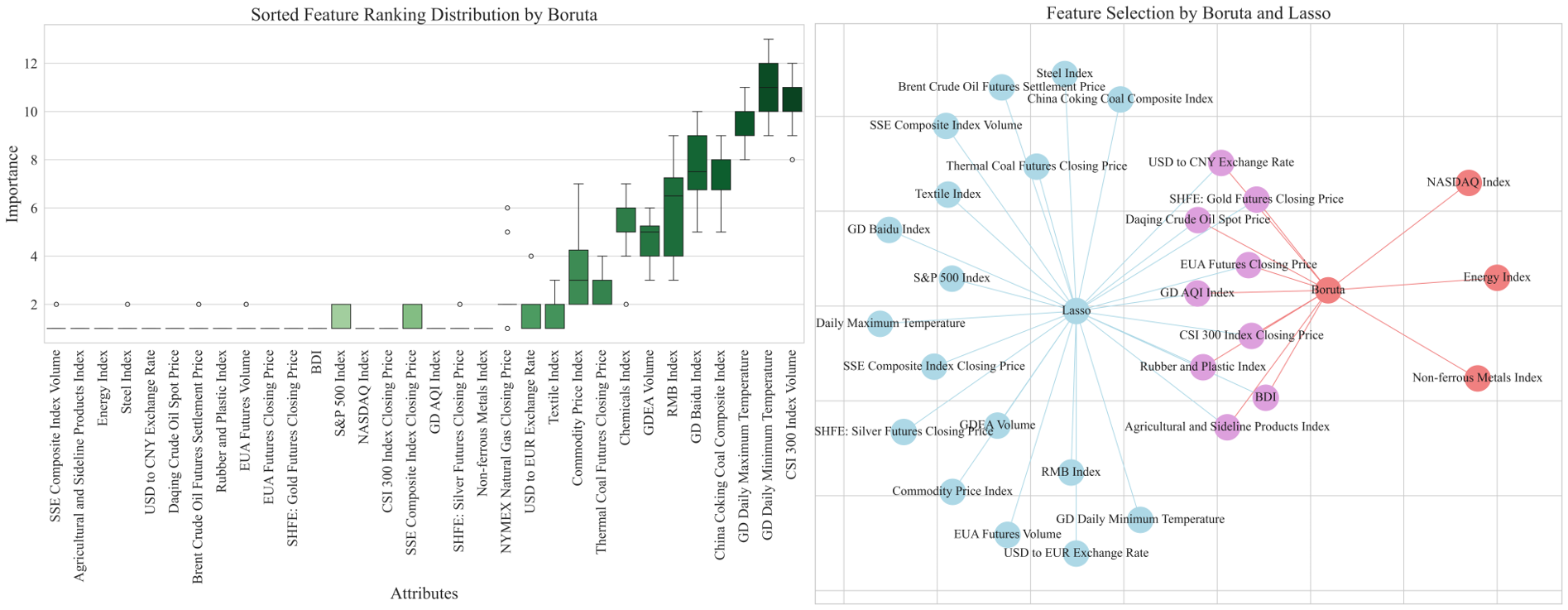

Supplement: S1 Data — (ZIP) [file pone.0326926.s001.zip › supplementary information/supplementary information/High-Definition Original Images in the Manuscript/Fig 7.tif]

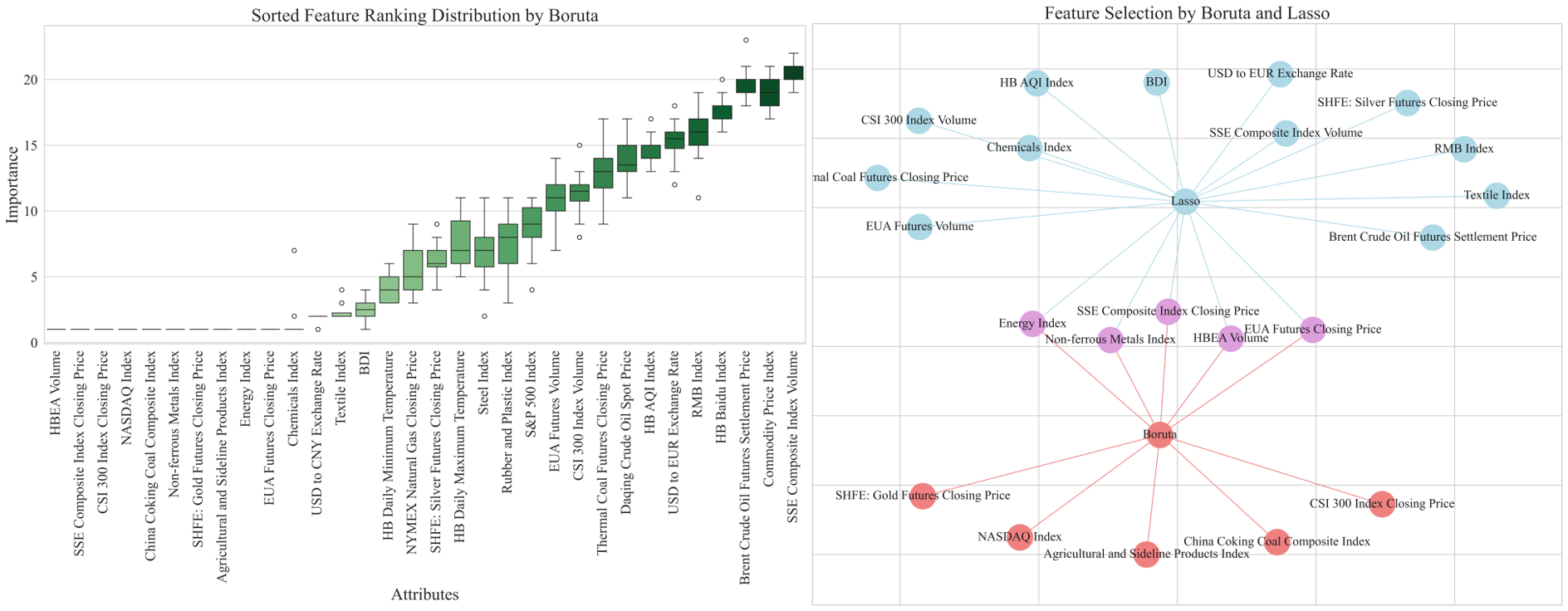

Supplement: S1 Data — (ZIP) [file pone.0326926.s001.zip › supplementary information/supplementary information/High-Definition Original Images in the Manuscript/Fig 8.tif]

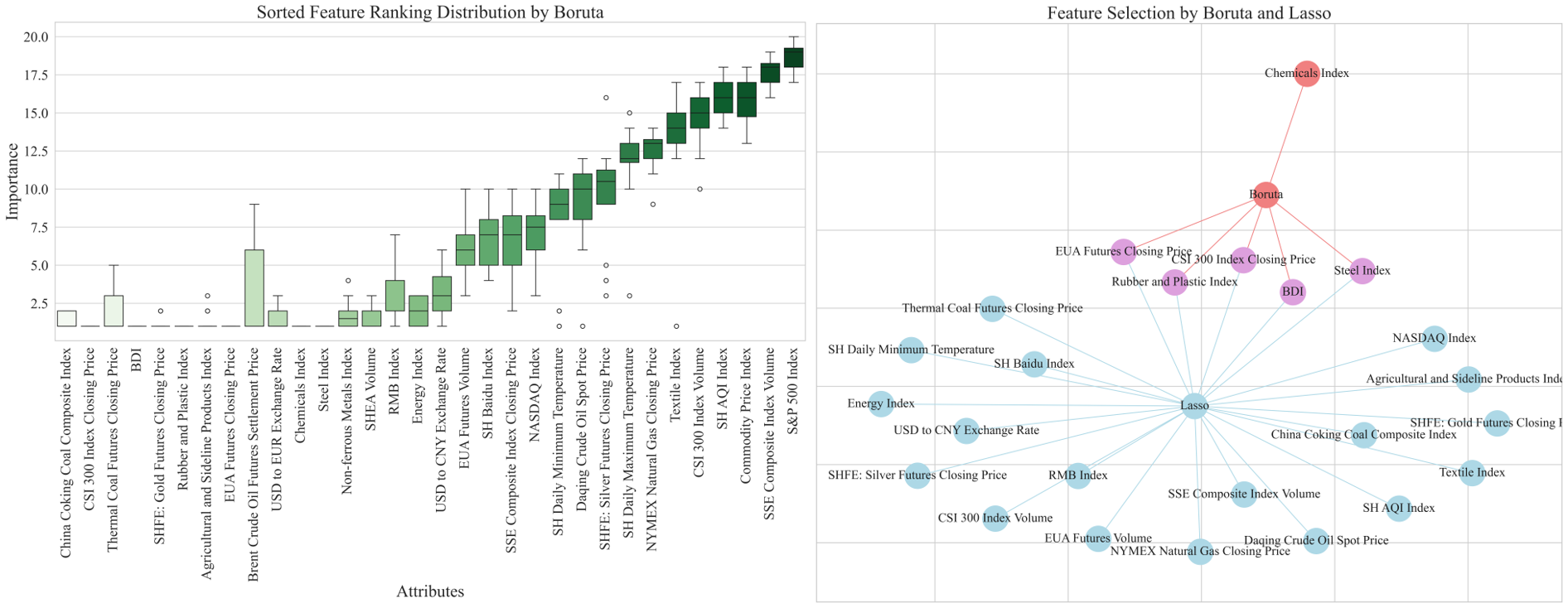

Supplement: S1 Data — (ZIP) [file pone.0326926.s001.zip › supplementary information/supplementary information/High-Definition Original Images in the Manuscript/Fig 9.tif]
